# A brain precursor atlas reveals the acquisition of developmental-like states in adult cerebral tumours

**DOI:** 10.1038/s41467-022-31408-y

**Published:** 2022-07-19

**Authors:** Akram A. Hamed, Daniel J. Kunz, Ibrahim El-Hamamy, Quang M. Trinh, Omar D. Subedar, Laura M. Richards, Warren Foltz, Garrett Bullivant, Matthaeus Ware, Maria C. Vladoiu, Jiao Zhang, Antony M. Raj, Trevor J. Pugh, Michael D. Taylor, Sarah A. Teichmann, Lincoln D. Stein, Benjamin D. Simons, Peter B. Dirks

**Affiliations:** 1 Department of Molecular Genetics, University of Toronto, Toronto, ON Canada; 2 Developmental and Stem Cell Biology Program, The Hospital for Sick Children, Toronto, ON Canada; 3 Arthur and Sonia Labatt Brain Tumour Research Centre, The Hospital for Sick Children, Toronto, ON Canada; 4 The Wellcome Trust/Cancer Research UK Gurdon Institute, University of Cambridge, Tennis Court Road, Cambridge, UK; 5 Cavendish Laboratory, Department of Physics, JJ Thomson Avenue, Cambridge, UK; 6 Wellcome Sanger Institute, Wellcome Genome Campus, Hinxton, UK; 7 Ontario Institute for Cancer Research, Toronto, ON Canada; 8 Department of Medical Biophysics, University of Toronto, Toronto, ON Canada; 9 Princess Margaret Cancer Centre, University Health Network, Toronto, ON Canada; 10 STTARR Innovation Centre, Department of Radiation Oncology, University Health Network, Toronto, ON Canada; 11 Department of Laboratory Medicine and Pathobiology, University of Toronto, Toronto, ON Canada; 12 Division of Neurosurgery, University of Toronto, Toronto, ON Canada; 13 Department of Surgery and Department of Medical Biophysics, University of Toronto, Toronto, ON Canada; 14 Department of Applied Mathematics and Theoretical Physics, Centre for Mathematical Sciences, Wilberforce Road, Cambridge, UK; 15 Wellcome Trust-Medical Research Council Stem Cell Institute, University of Cambridge, Cambridge, UK

**Keywords:** Neural stem cells, Cancer stem cells, CNS cancer

## Abstract

Human cerebral cancers are known to contain cell types resembling the varying stages of neural development. However, the basis of this association remains unclear. Here, we map the development of mouse cerebrum across the developmental time-course, from embryonic day 12.5 to postnatal day 365, performing single-cell transcriptomics on >100,000 cells. By comparing this reference atlas to single-cell data from >100 glial tumours of the adult and paediatric human cerebrum, we find that tumour cells have an expression signature that overlaps with temporally restricted, embryonic radial glial precursors (RGPs) and their immediate sublineages. Further, we demonstrate that prenatal transformation of RGPs in a genetic mouse model gives rise to adult cerebral tumours that show an embryonic/juvenile RGP identity. Together, these findings implicate the acquisition of embryonic-like states in the genesis of adult glioma, providing insight into the origins of human glioma, and identifying specific developmental cell types for therapeutic targeting.

## Abstract (web summary)

The link between neural development and tumourigenesis in adult glioma remains unclear. Here, the authors monitor the developmental stages of Sox2 + /− stem cells from a mouse model using single-cell RNA sequencing and suggest the acquisition of embryonic-like states in the adult glioma development.

## Introduction

The causes of primary brain tumours remain elusive with both the mechanisms and timing of initiation being poorly understood. Intra-tumoural heterogeneity in cerebral gliomas and glioblastomas (GBMs) have been extensively studied with several reports identifying cellular states that are reminiscent of the normal brain lineages^1–6^. However, these studies have not identified the linkages to developmental timing, lineage relationships or potential cells-of-origin in cerebral gliomas. In recent years, several reference maps of normal cerebral development have been derived. However, these maps are either incomplete, spanning restricted phases of development, or of limited depth and range, with precursor cells representing only a fraction of the sampled populations. Therefore, the potential link of normal developmental populations to tumours arising in the adult cerebrum has not yet been performed at scale and depth.

Several studies have sought to map human and mouse cerebral development at the single-cell level but, to date, these studies focused either on charting the embryonic/early postnatal phase of cortical development^7–10^, or on classifying neuronal and glial subtypes in the adult brain^11–18^. Moreover, the postnatal/adult brain studies were only able to retrieve a small population of precursor cells that represent only a minority of the total number of cells in the datasets, which were mostly comprised of mature neurons, astrocytes or immune cells^10,13–16,19^. Other studies used a targeted approach (e.g., using a stem cell reporter mouse line) to enrich for neural stem cell lineages^11,12,17,18,20–23^. Although they were successful in capturing the precursor cell populations and provided valuable insights into the aspects of adult neurogenesis, they only focused on the adult/aged stage of development and the abundance of neural precursors in these atlases were still limited.

Here, we aim to characterize the molecular identity of the precursors that may drive cerebral tumour cell heterogeneity, determining their potential relationships with normal developmental states and lineages, and their association to specific tumour types. We obtain a comprehensive mouse cerebral atlas that covers all stages of cerebral development and with the unique advantage of achieving a high resolution into the precursor compartments using the *Sox2eGFP* reporter mouse line. By examining molecular profiling data from over 100 patients, our results show that the diversity of adult cerebral tumour types associate closely with distinct developmental-like signatures, but not with later adult precursor populations. Further, to consolidate these findings, we perform a validation experiment showing that transformation of embryonic cerebral precursor cells using a transgenic mouse model gives rise to cellularly heterogeneous adult cerebral tumours that show an embryonic/juvenile RGP identity. Altogether, our results emphasize the ubiquitous role of normal developmental programmes in the growth and maintenance of cerebral tumours.

## Results

### A comprehensive single-cell atlas of precursor cells across cortical development

**Fig. 1:**
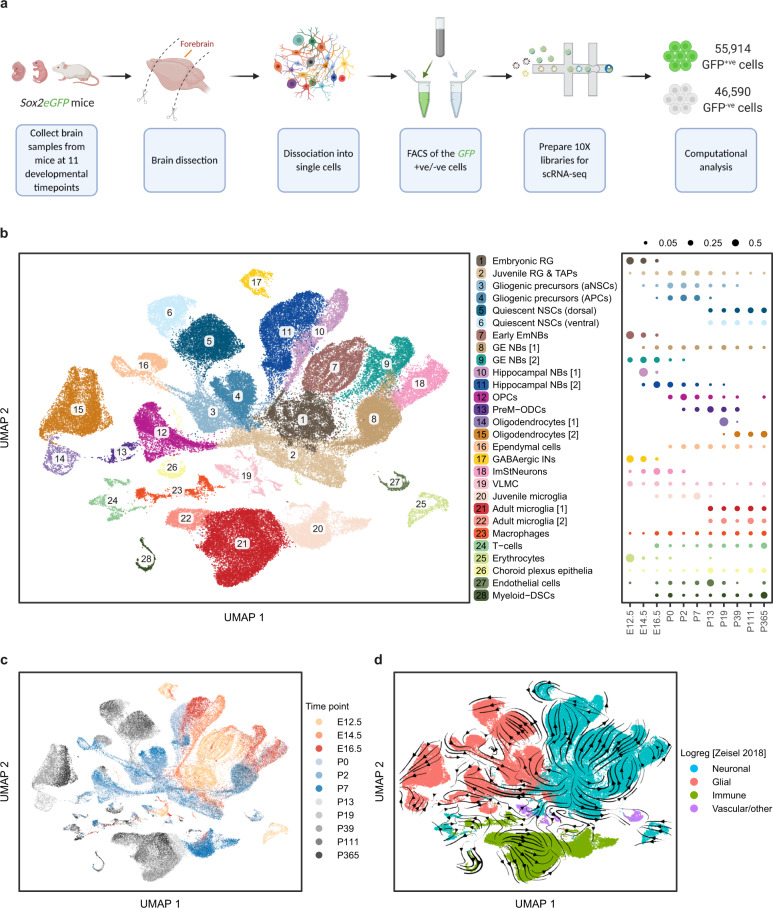
A comprehensive single-cell atlas of precursor cells across cortical development. **a** Schematic overview of the experimental workflow. Forebrain samples were collected from *Sox2eGFP* mice at 11 developmental timepoints, dissociated into single cells followed by sorting of the GFP + ve /−ve cells and performing single-cell RNA-seq using the 10x Genomics Chromium platform. **b** Left: two-dimensional Uniform manifold approximation and progression (UMAP) plot with Leiden clustering of 102,504 individual cells identifying 28 transcriptionally and biologically distinct cell types (see Supplementary Fig. 3a and Methods). Each dot represents a single cell and colours correspond to the distinct clusters/cell types. Shown on the right is a dot plot showing the relative fraction (dot size) of the 28 cell types at each developmental timepoint normalized by library size. **c** UMAP plot of the cells as in (**b**) but coloured by timepoint. Each dot represents a single cell and the colours correspond to the different timepoints. **d**, UMAP plot showing the coarse annotation of cells into 4 categories (Neuronal, Glial, Immune and Vascular/other) based on logistic regression from Zeisel et al. 2018. The RNA-velocity field is overlaid on the UMAP. Colours represent the 4 categories of cell annotation and the arrows correspond to the direction of the RNA-velocity flow field (see Methods). RG Radial glia, TAPs Transient amplifying progenitors, aNSCs Active neural stem cells, APCs Astrocyte progenitor cells, EmNBs Embryonic neuroblasts, GE NBs Ganglionic eminence neuroblasts, OPCs Oligoprogenitor cells, PreM-ODCs Immature pre-myelinating oligodendrocytes, INs Interneurons, ImStNeurons Immature striatal neurons, VLMC Vascular leptomeningeal cells, Myeloid-DSCs Myeloid-derived suppressor cells.

To characterize the cell types present in human cortical brain tumours, we first needed to define in depth the heterogeneity and lineage relationships of cells within the neurogenic and gliogenic compartments during the course of normal cerebrum development by applying single-cell RNA sequencing (scRNA-seq) and using the mouse brain as a model. Given that cerebral tumours are largely composed of precursor-like populations and contain few fully differentiated cells, we sought to develop a map that captures the normal precursor populations across the entire course of mouse cerebrum development. To do this, we made use of the transgenic *Sox2eGFP* reporter mouse model^24^. *Sox2* shows high expression in the cortical neurogenic zones across all stages of prenatal forebrain development and marking precursors in the geographically-restricted neurogenic and gliogenic zones of the postnatal and adult brain (Supplementary Fig. 1a). We collected cerebrum samples from *Sox2eGFP* mice at 11 timepoints spanning the key stages of brain development, from E12.5 to P365. Tissues were dissociated into single cells followed by fluorescence-activated cell sorting (FACS) of both the GFP^+^ and GFP^−^ cells to ensure roughly equal representation of the Sox2^+^ and Sox2^−^ cells (Supplementary Fig. 1b, c). scRNA-seq was then applied to the two groups of cells using the 10X Genomics Chromium platform (Fig. 1a). Following stringent quality controls, we obtained 102,504 single cells, with an average of 3,500 genes and 13,000 unique molecular identifiers per cell (Supplementary Fig. 2a, b).

Cells were found to segregate progressively into three main groups reflecting the embryonic (E12.5-E16.5), juvenile (P0-P7) and adult (P13-P365) stages of development (Fig. 1c and Methods). Consistent with the known pattern of lineage progression^25^, we identified large cell clusters within each group corresponding to four major categories: neuronal, glial, immune and vascular/other (Fig. 1d). This analysis revealed the predominance of neurogenesis during the earlier embryonic stage of development, followed by a switch to gliogenesis at around E16.5, which peaked during the juvenile stage and continued into the young adult stages (Fig. 1d, Supplementary Fig. 2c and Methods). Notably, RNA-velocity pseudotime analysis showed that the gliogenic compartment originates from neurogenic precursor cells present during the embryonic/juvenile stages (Fig. 1d and Methods). As expected, the majority of the GFP^+^/Sox2^+^ cells were comprised of neuronal and glial cells while most GFP^−^ cells were immune and vascular cells (Fig. 1d and Supplementary Fig. 2e). Unsupervised clustering of cells gave rise to 28 distinct clusters representing different lineages and cell types (Fig. 1b, Supplementary Figs. 2d, 3a and Methods). Annotating the clusters using known cell markers, we found that over 50,000 precursor cells and intermediate progenitors/neuroblasts were captured over the time course, constituting a comprehensive and detailed map of the key developmental stages of mouse cerebral development (Supplementary Figs. 2d, 3a).

### Temporal distribution of precursor cells throughout development

**Fig. 2:**
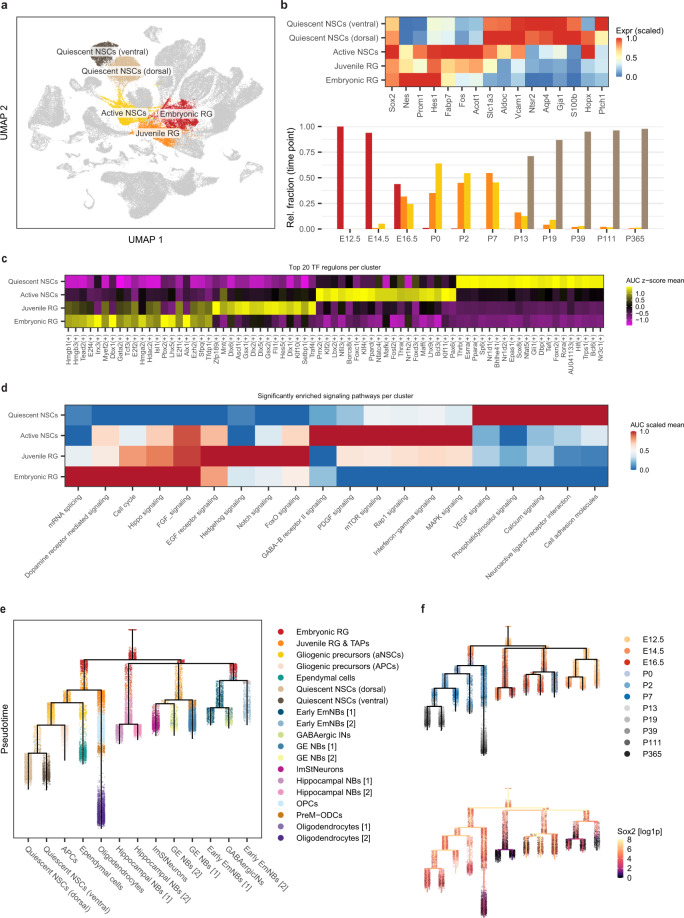
Temporal distribution of precursor cells throughout development. **a** Two-dimensional UMAP plot highlighting the main precursor populations identified across the 11 developmental timepoints. Each dot represents a single cell and colours correspond to the distinct precursor cell types. **b** Top: heatmap of the main marker genes used to differentiate/annotate the precursor clusters. Shown on the bottom panel is a bar plot showing the normalized relative fraction of the four precursor cell types grouped by timepoint. Colours correspond to the 4 distinct cell types (red: embryonic RG – orange: Juvenile RG – yellow: active NSCs – brown: quiescent NSCs). **c** Heatmap generated from the SCENIC analysis of the 4 precursor populations showing the top TF regulons per cluster. Colour scale indicates the mean regulon score. **d** A heatmap showing the significantly enriched signaling pathways in each of the 4 precursor populations. Colour scale indicates the scaled mean AUC score. **e** Lineage tree of the neuronal/glial cells within the atlas constructed using URD (see Methods). Each dot represents a single cell and colours correspond to the distinct clusters/cell types. **f** Top: lineage tree of the neuronal/glial cells within the atlas as in (**e**) but coloured by timepoint. Each dot represents a single cell and colours correspond to the different timepoints. Shown on the bottom is the log1p-expression of Sox2 overlaid on the lineage tree of the neuronal/glial cells. Each dot represents a single cell and the colour scale reflects the gene expression level. Source data are provided as a Source Data file. Abbreviations as in Fig. 1.

To gain insight into the diversity of precursor states during cortical development, we analyzed the precursor cells across the 11 timepoints. Unbiased clustering revealed four types of early precursor cells with distinct transcriptional profiles (Fig. 2a and Supplementary Fig. 3b). The first major cluster of cells were identified as embryonic RGPs, characterized by expression of the stem cell markers: *Sox2*, *Nes*, *Prom1* and *Hes1*^26–29^ (Fig. 2b). Embryonic RGPs were the dominant stem cell-like population at E12.5 and E14.5, while at E16.5, we observed the rise of a second population, which we termed juvenile RGPs (Fig. 2b). These cells expressed the same stem cell markers but were transcriptionally distinct from embryonic RGPs, as demonstrated by analysis of the differentially expressed (DE) markers (Supplementary Fig. 3b).

Contemporaneously with the switch from embryonic to juvenile RGPs, we identified a third population of progenitor cells that expressed the glial markers, *Slc1a3*, *Aldoc* and *Vcam1*^11,12,17,30^, as well as the known active neural stem cell (aNSCs) markers, *Fabp7*, *Fos* and *Acot1*^11,12,17^ (Fig. 2b). Based on marker expression and their temporal overlap with juvenile RGPs (Fig. 2b), we reasoned that this cluster represents the pool of early adult aNSC precursors.

Finally, in adult (P19-P365), the dominant stem cell populations were identified as quiescent neural stem cells (qNSCs), characterized by the expression of glial markers and the known qNSC marker, *Ntsr2*^12,22^, as well as the astrocyte markers *Aqp4*, *Gja1* and *S100b*^17,30^ (Fig. 2b). Interestingly, qNSCs clustered into two groups characterized by dorsal and ventral signatures (Fig. 2a, b).

To further characterize the differences between these temporally distributed precursor populations, we investigated the genetic and signaling networks that regulate their activity. We first examined the transcription factor (TF) regulons enriched in these clusters using SCENIC (Methods), which revealed an enrichment of “High mobility group” TFs (*Hmgb1*, *Hmgb3* and *Hmga2*) in embryonic/juvenile RGPs, Krueppel-like factors (*Klf2* and *Klf4*) in aNSCs, and “Nuclear receptor subfamily 1 group d” TFs (*Nr1d1* and *Nr1d2*) in qNSCs (Fig. 2c). Further, pathway enrichment analysis revealed other notable differences: FGF and EGF signaling were enriched in embryonic/juvenile RGPs and in aNSCs (Fig. 2d). By contrast, VEGF signaling was mostly enriched in qNSCs, while PDGF signaling was more enriched in aNSCs (Fig. 2d).

Finally, to identify the lineage relationships and the origin of differentiated cell types and adult brain precursors, we constructed a lineage tree of the neuronal/glial cells within the atlas using URD^31^, a computational method that constructs a lineage tree based on the pseudotime trajectory and transcriptional similarity of the cells (Methods). Based on previous findings from the RNA-velocity analysis (Fig. 1d) and their expression pattern at the earliest timepoint, we defined the root of the tree as the E12.5 embryonic RGPs. This analysis placed embryonic/juvenile RGPs at the origin of multiple sublineages including neuroblasts, oligodendrocyte progenitor cells (OPCs), astrocyte progenitor cells (APCs) and ependymal cells, as well as aNSCs which, in turn, populate qNSCs in adult (Fig. 2e, f). Interestingly, *Sox2* was significantly expressed across most branches of the tree and was partially but not completely downregulated in the neuroblast and oligodendrocyte branches, as evidenced by the residual expression of GFP in these populations (Fig. 2f and Supplementary Fig. 3c).

### Regional distribution of radial precursors during the early embryonic stage

**Fig. 3:**
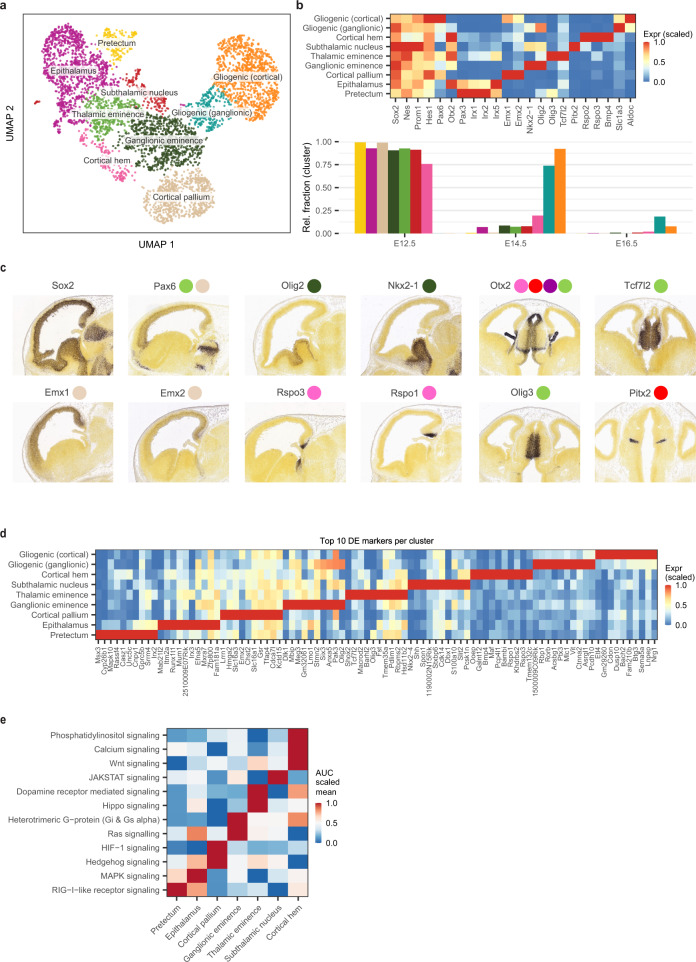
Regional distribution of radial precursors during the early embryonic stage. **a** UMAP plot with Leiden clustering of the embryonic RG cells showing the main clusters/cell-subtypes identified. Each dot represents a single cell and the colours correspond to the different clusters. **b** Top: heatmap of the main marker genes used to annotate the clusters in (**a**). Shown on the bottom panel is a bar plot showing the normalized relative fraction of the distinct clusters grouped by timepoint. Colours correspond to the clusters in (**a**). **c** In-situ hybridization staining of sagittal (columns #1-4) and transverse (columns #5, 6) slices from E13.5 mouse embryos, showing the expression of some of the markers used to identify the clusters in panel (**a**). In-situ hybridization images were obtained from the Allen Brain Map: Developing Mouse Brain Atlas. The coloured circles correspond to the clusters in panel (**a**). **d** Heatmap of the top 10 DE marker genes identified per cluster from analyzing the clusters in panel (**a**). Colour scale indicates the scaled mean expression in each cluster. **e** A heatmap showing the significantly enriched signaling pathways in each of the regional embryonic RG subtypes. Colour scale indicates the scaled mean AUC score. Source data are provided as a Source Data file. Abbreviations as in Fig. 1.

We next analyzed the cell cycle characteristics to define subpopulations within the four major precursor cell types and then confirmed these differences by performing unsupervised clustering on the isolated cells. Of note, when applied to the embryonic RGPs, both techniques identified a set of nine distinct subgroups of cells (Fig. 3a and Supplementary Fig. 3d). Further examination of these clusters revealed that seven (found predominantly at E12.5) showed expression signatures linked to their geographical location: *Emx1, Emx2* and *Pax6* (cortical pallium)^32,33^, *Rsop2*, *Rspo3* and *Bmp4* (cortical hem)^34,35^, *Nkx2-1* and *Olig2* (ganglionic eminence)^36,37^, *Tcf7l2*, *Olig3* and *Pax6* (thalamic eminence)^38–40^, *Irx1*, *Irx2* and *Pax3* (pretectum)^38,41^, *Irx1*, *Irx2* and *Otx2* (epithalamus)^38,41^, and *Otx2* and *Pitx2* (subthalamic nucleus)^42^ (Fig. 3b). The markers used to signature the distinct spatial subtypes of embryonic RGPs were validated by examining their expression patterns in the developing mouse brain from the Allen Mouse Brain Atlas (Fig. 3c) (https://developingmouse.brain-map.org/). At E14.5, two further clusters emerged that expressed the glial markers, *Aldoc* and *Slc1a3*, suggesting that these cells represent early gliogenic RGPs (Fig. 3b). Interestingly, one of the gliogenic clusters expressed the cortical pallium markers (*Emx1* and *Emx2*) while the other expressed the ganglionic eminence markers (*Olig2*, *Nkx2-1* and *Ptch1*) indicating their distinct regional identity/origin (Fig. 3b).

To further identify the expression signatures that distinguish the embryonic RGP subtypes, we generated a list of the top DE markers between clusters (Fig. 3d), as well as the relative enrichment of TF regulons and signaling pathways (Fig. 3e and Supplementary Fig. 3e). This analysis revealed notable differences between the clusters: For example, Hippo signaling was enriched in the thalamic eminence precursors, Hedgehog and Hif1 signaling were enriched in the cortical pallium precursors, Wnt and calcium signaling were enriched in the cortical hem precursors, and Ras signaling was enriched in the epithalamus and ganglionic eminence precursors (Fig. 3e). These results identify a series of region-specific subtypes of embryonic RGPs during the early stage of cerebral development.

### Temporal diversity of the lineage cell-types throughout forebrain development

We next examined the distribution of sublineages across the distinct developmental stages. We identified several groups of early embryonic neuroblasts (EmNBs), which were present exclusively during embryonic stages E12.5-E14.5 (Supplementary Fig. 4a), consistent with the known temporal restriction of cortical neurogenesis^25^. These cells were marked by the expression of *Nhlh2*, *Nhlh1* and *Ebf2*^43,44^, distinguishing them from juvenile/adult NBs (Supplementary Fig. 4b, c). At later timepoints, we identified clusters of OPCs and ependymal cells, which were largely restricted to the juvenile stages and were transcriptionally distinct from the corresponding adult clusters (Supplementary Fig. 5d–h).

Together, these results define multiple layers of temporal and regional restricted major precursor populations as well as developmentally restricted lineage cell-types throughout cerebral development. To make the data readily accessible, we have prepared an App (https://shiny-server.gurdon.cam.ac.uk/djk44/brain-development_app/) that allows the atlas and lineage relationships to be freely explored.

### Adult and paediatric human GBMs show an embryonic/juvenile-like cell signature

Armed with a detailed atlas of mouse cerebral development, we then sought to identify the putative cell types and sublineages that comprise adult and childhood cerebral tumours. To do this, we used CIBERSORTx, a deconvolution algorithm that allows mapping of the human scRNA-seq data to the mouse clusters (Methods)^45,46^. This regression-based partial gene expression method reveals the relative abundance of the normal cell clusters in tumour transcriptomic data^47–49^, with the advantage of including a large number of gene signatures (our signature matrices vary from 2000 to 4000 genes) and takes into account the varying gene expression levels. This approach allowed us to predict the relative contribution of all the cell-type-specific signatures (from the reference cell-type dataset, as defined by the mouse atlas) that is present in each tumour population. Importantly, by preparing a reference atlas that spans the entire developmental time course, we could be confident in identifying all potential cell type and lineage associations from the tumour datasets.

To control for batch effects, and to test the fidelity of cross-species comparisons, we first asked whether single-cell data from normal human foetal cortices at gestation week 17-18^50^ could be aligned with the cellular subpopulations from the mouse atlas. This mapping revealed strong similarities between human foetal RGPs and the mouse embryonic and juvenile RGPs (Supplementary Fig. 5c). Further similarities were evident among several lineages, including OPCs and ganglionic eminence NBs (Supplementary Fig. 5c). Together, these results provided confidence that, for normal tissue, cell types can be matched accurately across human and mouse cerebral precursor populations.

Based on the reliability of the cross-species mapping, we then analyzed published scRNA-seq data from human IDH-wildtype GBMs from adult and paediatric patients^1,51^, comparing the transcriptome of clusters from each patient to our mouse atlas. To focus the analysis on precursor cells and the neuronal/glial lineages, we excluded immune cells from both the mouse and human datasets and re-clustered the mouse data (Supplementary Fig. 5a). Further, to eliminate potential bias in the matching, we excluded gene sets associated with common biological processes from the deconvolution analysis, including cell cycle and apoptosis-related genes (Methods).

**Fig. 4:**
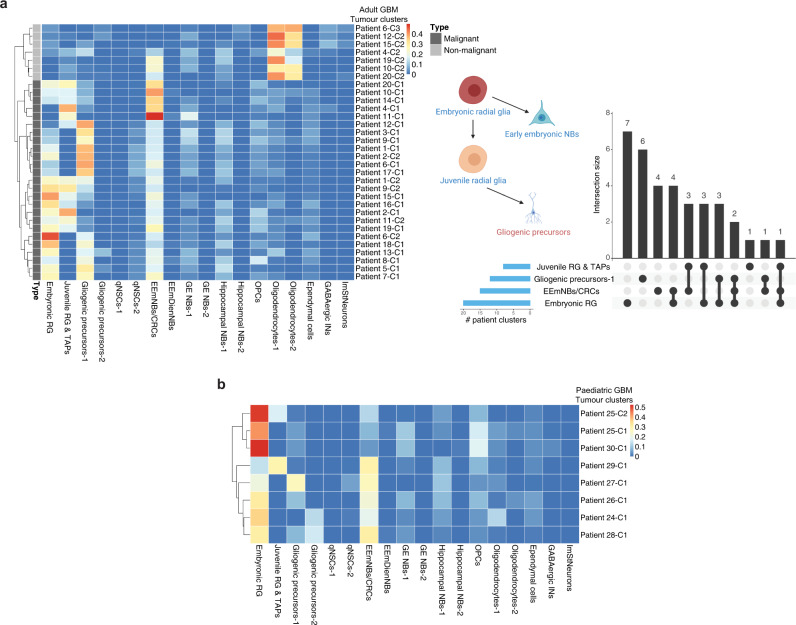
Adult and paediatric human GBMs show an embryonic/juvenile-like cell signature. **a** Left: deconvolution analysis heatmap of malignant and non-malignant clusters from 20 adult GBM patients from Neftel et al. 2019 showing the estimated relative fraction of the mouse cell-types for each patient cluster (see Methods). The bar on the left indicates the state of the cells (malignant or non-malignant). Each patient sample was clustered separately (see patient clustering in Supplementary Fig. 5d) and the clusters are denoted as e.g., “Patient#1-cluster#1” = “Patient 1-C1” (see Supplementary Data 1). Shown on the right is a bar plot summarizing the matching results and overlap size between the patient clusters (e.g., 3 patient clusters matched to both embryonic RG and gliogenic precursors) and a schematic summary of the tumour lineage from the deconvolution analysis. **b** Deconvolution analysis heatmap of malignant clusters from 7 paediatric GBM patients from Neftel et al. 2019 (see Supplementary Data 1) showing the relative fraction of the mouse cell-types for each patient cluster. Each patient sample was clustered separately (see patient clustering in Supplementary Fig. 6a) and the clusters are denoted as e.g., “Patient#24-cluster#1” = “Patient 24-C1” (see Supplementary Data 1). RG Radial glia, TAPs Transient amplifying progenitors, qNSCs Quiescent neural stem cells, EEmNBs/CRCs Early embryonic neuroblasts/Cajal-Retzius cells, EEmDienNBs Early embryonic diencephalon neuroblasts, GE NBs Ganglionic eminence neuroblasts, OPCs Oligoprogenitor cells, INs Interneurons, ImStNeurons Immature striatal neurons.

Neoplastic cells from both childhood and adult GBM samples showed high similarity to neural precursor cell types, showing a particular enrichment for signatures of mouse embryonic and juvenile RGPs, as well as EmNBs (Fig. 4a, b and Supplementary Fig. 8a). By contrast, we found no significant overlap with adult qNSCs, nor adult-restricted lineage cell types (e.g., adult GE and hippocampal NBs). Some clusters in individual patient tumours matched closely with gliogenic progenitors, reflecting a glial/astrocytic lineage, consistent with the long-held view of GBM cell architecture^52^, and suggestive of a differentiation process that extends from embryonic RGPs to gliogenic precursors (Fig. 4a and Supplementary Fig. 8a). Together, these findings suggest that both juvenile and adult human GBMs are transcriptionally most similar to normal cells with an embryonic and early postnatal-restricted cell identity.

### Identifying the genes shared between the GBM cells and the matching embryonic precursors

Next, to assess the extent of the shared transcriptional identity, we generated a list of the top genes shared between the individual tumour clusters in the GBM patient samples and the matching mouse embryonic RGPs (Supplementary Fig. 8b and Supplementary Data 2). Surprisingly, we found that these included genes that are neither well known, nor reported, in GBM studies. However, notably, many of these genes have been reported as essential for the function of cancer cells in other contexts. For example, the copper chaperone ATOX1 plays an essential role in the migration of breast cancer cells^53^ and silencing the activity of the GTPase RAB2A inhibits the growth of breast cancer stem cells^54^. Similarly, the ubiquitin-conjugating enzyme UBE2V2 is required for the growth and proliferation of melanomas^55^. If confirmed, these genes may correspond to targetable pathways for GBM therapeutics.

### Cerebral gliomas show developmental identities that reflect a foetal-like nature

**Fig. 5:**
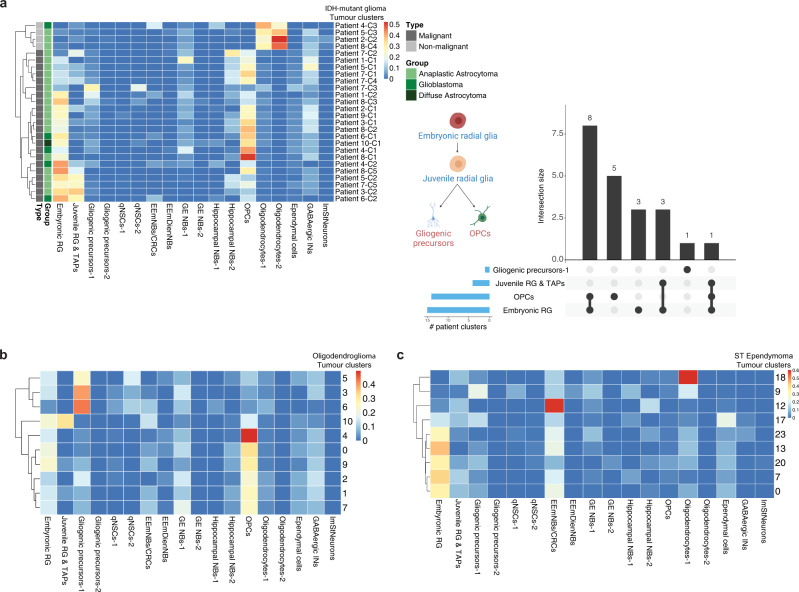
Cerebral gliomas show developmental identities that reflect a foetal-like nature. **a** Left: deconvolution analysis heatmap of tumour clusters from 10 IDG patients from Venteicher et al., 2017 showing relative fractions of mouse cell-types for each patient cluster. Bars on the left indicate the state of the cells (malignant or non-malignant) and the type of glioma. Each patient sample was clustered separately (see patient clustering in Supplementary Fig. 7) and the clusters are denoted as e.g., “Patient#1-cluster#1” = “Patient 1-C1” (see Supplementary Data 1). Shown on the right is a bar plot summarizing the matching results and overlap size between the patient clusters and a schematic summary of the tumour lineage from the deconvolution analysis. **b** Deconvolution analysis heatmap of tumour clusters from 6 ODG patients from Tirosh et al. 2016 showing relative fraction of the mouse cell-types for each tumour cluster. The patient samples were analysed and clustered together (see Methods and clustering analysis in Supplementary Fig. 9b). **c** Deconvolution analysis of scRNA-seq tumour clusters obtained from four paediatric supratentorial ependymomas showing relative fraction of the mouse cell-types for each tumour cluster. The patient samples were clustered together and immune cells were identified and excluded using the expression of known immune markers (see Methods and clustering analysis in Supplementary Fig. 7d, e). Abbreviations as in in Fig. 4.

We next considered cell type identities in other types of gliomas prevalent in mostly younger adults, including IDH-mutant gliomas (IDG) and oligodendrogliomas (ODG). Comparing scRNA-seq data from 10 IDG^4^ and 6 ODG^3^ patients to the developmental mouse atlas, we found that tumour clusters had a high similarity to embryonic/juvenile RGPs (Fig. 5a, b). However, unlike most of the IDH-wildtype GBMs, we did not find overlap with early EmNBs, consistent with the absence of neuronal lineage potential in these tumours^3,4^, while several clusters showed high similarity to mouse OPCs (Fig. 5a, b). Further clustering of the mouse OPCs showed that the IDG/ODG patient clusters map more closely with juvenile rather than adult mouse OPCs (Supplementary Fig. 9a, c), which further confirmed the embryonic/juvenile-like nature of these tumours.

We also examined scRNA-seq data from four supratentorial ependymomas, a paediatric glioma that accounts for nearly 30% of ependymomas. Mapping to the mouse developmental atlas revealed a similar pattern of tumour heterogeneity to that found in the IDH-wildtype GBMs, with high similarity to embryonic RGPs as well as to EmNBs (Fig. 5c).

To further validate these findings, we matched the human foetal scRNA-seq data^50^ to tumour clusters from the IDH-wildtype GBM and IDG datasets. Consistently, clusters that showed high similarity to mouse embryonic RGPs and OPCs also matched human foetal RGPs/OPCs (Supplementary Fig. 7f–h), bolstering our confidence in the ability of the mouse atlas to characterize cellular heterogeneity in cerebral tumours across the species divide.

### Tumour-derived cell lines are enriched for embryonic RG-like cells

**Fig. 6:**
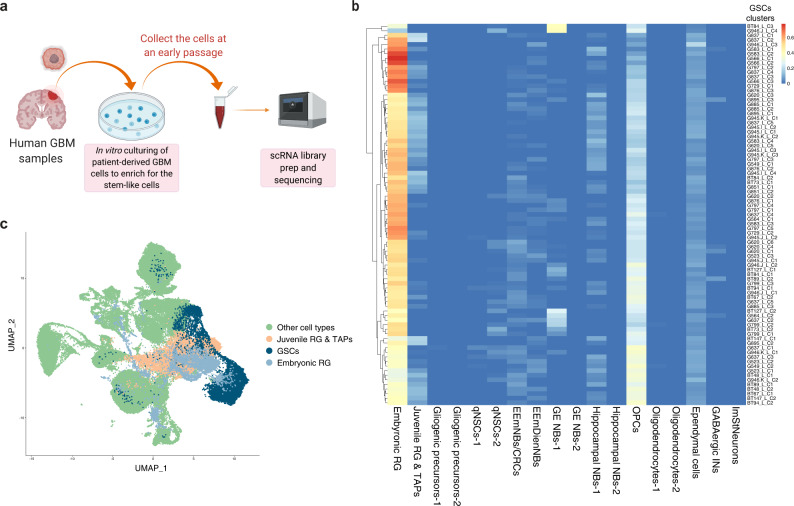
GBM patient-derived cell lines are enriched for embryonic RG-like cells. **a** Overview of the experimental workflow for the collection of GBM stem cells (GSCs). **b** Deconvolution analysis heatmap of the GSCs clusters obtained from the scRNA-seq of 26 patient-derived GBM cultures from Richards et al. 2021 showing relative fraction of the mouse developmental cell-types for each GSC cluster. Each patient GSCs sample was clustered separately and the clusters are denoted as e.g. “G523_Line_cluster#1” = “G523_L_C1”. **c** Co-clustering of the GSCs obtained from the scRNA-seq of 26 patient-derived GBM cultures with the clusters from the mouse developmental dataset (see Methods). Abbreviations as in Fig. 4.

GBMs are thought to originate from renewing stem cell-like populations that have both the capacity to survive in serum-free cell culture conditions and, when xenografted, can produce a tumour that phenocopies the original patient tumour^56^. To gain insight into the cellular and molecular identity of these tumour-initiating subpopulations, we used scRNA-seq data generated from 26 patient-derived GBM cultures^51,57–59^ (Fig. 6a). Strikingly, the deconvolution analysis revealed that the majority of the cells in primary cultures matched closely with the mouse embryonic RGPs (Fig. 6b). These conclusions were further strengthened by co-clustering the mouse developmental atlas with the scRNA-seq data from the patient-derived cell lines (Methods), which showed that the patient cells co-clustered with the mouse embryonic RGPs (Fig. 6c). These findings suggest that primary culture conditions support the maintenance of a dominant embryonic-like precursor cell type in GBM, potentially reflecting the identity of the tumour-maintaining subpopulations in vivo^56^.

### Transformation of the prenatal mouse cerebral precursors gives rise to adult cerebral tumours that show an embryonic/juvenile RGP identity

**Fig. 7:**
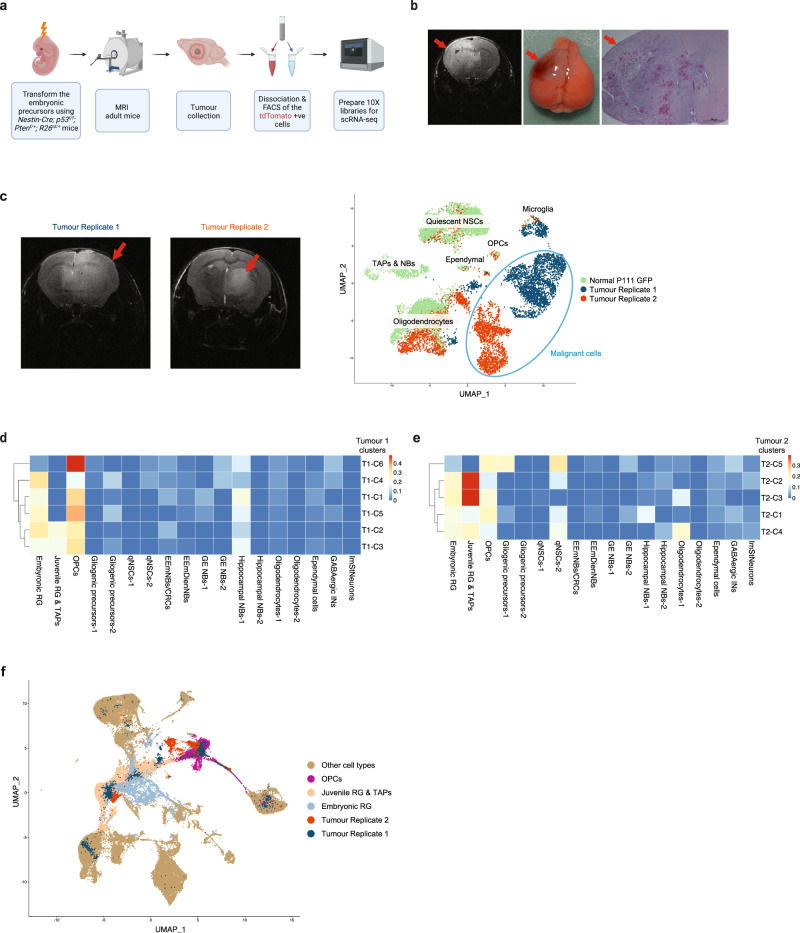
Transformation of the prenatal mouse cerebral precursors gives rise to adult cerebral tumours that show an embryonic/juvenile RGP identity. **a** Schematic overview of the experimental workflow. **b**, MRI of a mutant mouse brain at 16 weeks of age showing a tumour encompassing the left hemisphere (red arrows) with an image of the brain after dissection and an H&E-stained section. The brain was collected 30 min after the MRI. Scale bar, 100 µm. **c** Left: mouse brain MRI from two tumour replicates, the scans were performed 30 min before brain sample collection. Shown on the right is the UMAP clustering of single-cell transcriptomes from the two mouse tumour replicates and the GFP^+^ cells from the P111 timepoint in the developmental atlas. **d**, **e** Deconvolution analysis heatmap of the malignant clusters from tumour replicate 1 (**d**) and tumour replicate 2 (**e**), showing relative fraction of the mouse developmental cell-types for each tumour cluster (see Methods). The malignant cells from each tumour replicate were clustered separately (see Supplementary Fig. 10b, c and Methods) and the clusters are denoted as e.g., “Tumour-replicate#1 cluster#1” = “T1-C1”. **f** Co-clustering of the cells from the two tumour replicates with the clusters from the mouse developmental dataset (see Methods). Abbreviations as in Fig. 4.

Finally, to validate the existence of RGPs in adult cortical neoplasia, we used the *Nestin-Cre* mouse model to drive *Cre* expression in vivo in cells that endogenously express *Nestin*, allowing us to activate oncogene expression in prenatal precursors in which *Nestin* is highly expressed at E12.5 (Supplementary Fig. 3a). We generated the *Nestin*^*Cre/+*^; *p53*^*f/f*^; *Pten*^*f/+*^; *R26*^*tdTomato/+*^ tumour model in which *Cre*-mediated recombination results in the activation of the *tdTomato* reporter as well as the homozygous and heterozygous deletion of the two tumour suppressors, *Tp53* and *Pten*, respectively, genes that are mutated/deleted in many GBM patients^60^. These mice develop anaplastic astrocytomas and GBMs by 12-36 weeks of age (Fig. 7a, b and Supplementary Fig. 10a). Magnetic resonance imaging (MRI) of the mice allowed us to confirm the presence and size of tumours in the cerebrum (Fig. 7b and Supplementary Fig. 10a). Once they reached an endpoint stage, tumours were removed, dissociated into single cells and FACS for *tdTomato* expression, before scRNA sequencing using the 10X platform (Fig. 7a and Supplementary Fig. 10d). We obtained a total of 6798 cells from two tumour replicates (collected at P112 and P108). To distinguish malignant and non-malignant cells within the two tumour samples, we clustered the tumour cells with normal GFP^+^ cells from the P111 timepoint in the developmental atlas (chosen as the closest timepoint to the age of the tumour mice). As expected, non-malignant cells intermixed with P111 cells, while the malignant cells clustered separately (Fig. 7c).

Next, we used deconvolution analysis to map the malignant cells to the mouse developmental atlas to resolve the identity of these cells. The analysis showed that malignant cells from both replicates had a high similarity to embryonic/juvenile RGPs, indicating either a persistence of these early lineage states following oncogene activation or their reactivation during tumourigenesis (Fig. 7d, e). Several clusters from both replicates also showed high similarity to OPCs (Fig. 7d, e) indicating an OPC lineage in these tumours, which we further validated by staining tumour tissue for the OPC marker Pdgfra (Supplementary Fig. 11a, b). Consistently, co-clustering the clusters from the two mouse tumour replicates with the normal mouse atlas clusters (Methods) showed that tumour cells co-clustered with embryonic/juvenile RGPs and OPCs (Fig. 7f), providing further validation of the deconvolution analysis and implicating the acquisition of developmental-like states in the genesis of adult glioma.

## Discussion

By mapping the gene expression profiles of individual Sox2^+^ and Sox2^−^ cells in the mouse cerebrum across the entire developmental time course, from the early neurogenic phase of embryonic development, through the gliogenic phase and into adult, we have obtained a single-cell atlas detailing the sublineage relationships, regional identities and transcriptional changes that take place during cortical development, with a focus on the precursor cell types. Although previous studies have sought to map mouse brain development at the single-cell level, to date, the majority have focused either on charting the embryonic/early postnatal phase of cortical development, or on classifying neuronal and glial subtypes in the adult brain. However, to determine the potential association of cancer cells with cell types found during normal brain development, it is essential to compare tumour data with a map of precursor states that spans the entire developmental time course. Further, to understand whether and how cancer cells map to distinct normal precursor subtypes, it is necessary to achieve a high level of resolution, calling for a targeted approach in which the precursor cell fraction of sampled cells is enriched. Therefore, we used a reporter-based approach to develop a comprehensive map of cortical development, from embryo to adult, enriching for the ensemble of Sox2^+^ precursors and their progenies. By combining GFP^+^ and GFP^−^ cells across all timepoints, we could ensure a broad coverage of proliferative and differentiated cell types. Altogether, our atlas comprises data from 11 developmental timepoints, capturing a total of 55,914 GFP^+^ and 46,590 GFP^−^ cells. Crucially, in contrast to recent studies that avoided using a reporter mouse model or sorting for stem cell markers, we were able to capture major fractions of precursor cells spanning all stages of the mouse brain development. At this resolution, we were able to map the temporal distribution of various precursor cell types and their immediate sublineages, providing a detailed expression signature to resolve cancer cell type identities.

As well as constituting a unique valuable resource, the atlas served as a reference to map the molecular cell identities and sublineage relationships across the wide range of human brain tumour subtypes that arise in the cerebrum. Despite evidence of intra- and inter-tumoural heterogeneity^1,2,61^, our results suggest that the growth of both paediatric and adult brain tumour subtypes involve the aberrant activation of normal foetal developmental programmes that dominate tumour cell composition.

The association of adult GBM cells with a RGP-like signature has been proposed in the literature based on scRNA-seq analyses^62–64^. However, GBM tumour cells are known to have properties reminiscent of both the embryonic and adult precursors, and so the individual comparison of tumour cells to either embryonic or adult precursors will reveal similarities to both. Therefore, only a comparison of tumour data with a comprehensive developmental time course can accurately discriminate between the developmental RGPs and the adult NSC-like identity. By examining data from over 100 patients, belonging to different types of glial tumours and not just GBMs, our study revealed that, despite the phenotypic/genotypic differences between the different tumour types, they are all comprised of distinct stage-specific developmental sublineages that map most closely to embryonic and/or juvenile stages of development; and not adult. The foetal-like program is reflected in the matching to several embryonic/juvenile lineages including the embryonic/juvenile RGPs, the embryonic-restricted neuroblasts as well as the juvenile OPCs, suggesting either a point of origin for tumors at earlier stages in development or activation of earlier programmes in potentially more differentiated cells of origin. Together, these findings emphasize the ubiquitous role of normal developmental programmes in the maintenance of tumour growth.

We acknowledge the potential limitations of CIBERSORTx, but we believe that our experimental design addressed these limitations. A regression-based partial gene expression method, such as CIBERSORTx, reveals the relative abundance of the normal cell clusters in tumour transcriptomic data. Close inspection of the results showed that most individual tumour populations match to multiple cell-type-specific signatures, shown by the relative abundance scores. This approach allowed us to predict the relative contribution of all the cell-type-specific signatures (from the reference cell-type dataset, as defined by the mouse atlas) that is present in each tumour population. Therefore, it was important that the reference cell-type dataset (mouse dataset) includes all the possible lineages that are expected to be present in the tumour datasets. Indeed, this is why it was essential to build an atlas that spans the entire developmental time course, from the embryonic neurogenic phase to the late embryonic/juvenile gliogenic phase and on into adult neurogenic phases. Moreover, by virtue of their relative scarcity, particularly in the postnatal mouse brain, to achieve a high level of resolution, we made use of a Sox2-sorting approach in which the precursor cell fraction (GFP^+^/Sox2^+^) of sampled cells is enriched, while GFP^−^/Sox2^−^ cells were also collected and profiled to ensure a representative map of all cell types.

Our findings raise the question of whether the initiation of adult glioma involves the transformation of precursor cells that failed to complete their normal differentiation programme during development, or whether they derive from the “reprogramming” of mature cell types in the adult. To begin to address this question, we made use of an existing genetic mouse model in which driver mutations were activated in embryonic precursor cells. Our results showed that the mouse tumour malignant cells had a high similarity to embryonic/juvenile RGPs, indicating either a persistence of these early lineages following oncogene activation or their reactivation during tumourigenesis. They also raise important questions as to how such tumours initiate, particularly in the adult brain milieu, which is largely devoid of proliferation and regenerative capacity.

The conservation of developmental sublineages identities within tumour subtypes suggests a hierarchical organization^65^, providing a framework to identify the molecular and functional identity of tumour-maintaining populations and their specific progenies that comprise tumour bulk, and raising implications for designing treatment strategies that target the full tumour cellular heterogeneity. The findings of this study echo the results of molecular characterizations in other tumour models, including adult cancers, that have identified the key role of normal developmental programmes in early tumour progression^66^. Our work identifies transient foetal populations as the best transcriptional match for adult GBM tumours. One possible interpretation of this finding might be that the tumour precursor arises during human foetal development but persists and lies dormant until adulthood. However, this seems unlikely, particularly in light of evolutionary models of cancer whole genome sequencing data that project a median time between initiation and diagnosis of GBM of several years at most^67^. An alternative explanation is that the tumour arises in a more mature adult cell that dedifferentiates during the process of malignant transformation. Distinguishing between these two possibilities will have implications for future research and clinical management. In particular, discerning the timing and mechanisms of acquisition of the embryonic state will have important implications for considering the causes and subsequent evolution of primary brain tumours. A key experiment will be to explore the early stages of GBM development in genetic mouse models through scRNA-seq of the malignant cells existing within the preneoplastic and early lesion stages of tumour development as guided by MRI.

Importantly, as well as providing molecular targets for early detection, the association of tumour growth with the activation of developmental programmes may suggest intervention strategies that target differentiation rather than cell proliferation. It also should lead to a greater precision of targeting the heterogeneous cell types that comprise specific childhood and adult cerebral tumours.

## Methods

### Animal experiments

All mouse experiments were approved by the Hospital for Sick Children’s Animal Care Committee and following all legal and institutional ethical regulations. *Sox2eGFP* (*Sox2*^*tm1Lpev*^) mice^24^ were provided by Dr. Freda Miller, Toronto, Hospital for Sick Children. *p53*^*f/f*^ mice^68^ were provided by Dr. Chi-chung Hui, Toronto, Hospital for Sick Children. The following transgenic strains were purchased from Jackson Laboratory: *Nestin-Cre* (JAX# 003771), *R26*^*td/td*^ (B6.Cg-Gt(ROSA)26Sor^tm9(CAG-tdTomato)Hze^/J) (JAX #007909) and *Pten*^*f/f*^ (JAX# 006440). The mice were housed in a 12 h dark/light cycle facility with free access to water and chow. The mutant mice that are expected to get tumours were monitored daily and were euthanized once they show endpoint symptoms (e.g. domed head, dehydration, ataxia…etc).

### Human tissue collection

The human tissue was collected from the Hospital for Sick Children Brain Tumour Tissue Bank after patient consent for banking. The samples were de-identified and the research conducted was performed following informed written consent from the patients’ parents or guardians and approval from the Research Ethics Board of the Hospital for Sick Children. The human supratentorial ependymoma tissue was processed for scRNA-sequencing as previously reported^49^. Fresh tumor tissue was collected at the time of resection and enzymatically dissociated using collagenase-dispase dissociation method. Cells in a single cell suspension were counted via trypan blue and loaded on the Chromium controller.

### Dissection and processing the mouse brain samples for single-cell sequencing

Fresh brain tissues from embryo/postnatal *Sox2eGFP* mice were collected following intracardial perfusion of mice (pregnant females in case of embryo samples) with PBS. The tissue was transferred to a petri dish placed on ice and the cerebrum was isolated under a Leica stereoscope, rinsed in PBS, dissociated into single cells through mechanical dissociation and, when needed, enzymatic dissociation (using Accutase), then passed through a 40-µm mesh cell strainer. Debris was removed using a debris-removal kit (Miltenyi) and the cells were stained with DAPI followed by sorting the live GFP^+ve/−ve^ cells. For the mouse tumour samples, the mouse harboring the tumour was imaged by MRI 30 min before sample collection through intracardial perfusion with PBS and dissection of the hemisphere encompassing the tumour followed by tissue dissociation as described above. Finally, the cells were stained with DAPI before sorting the live tdTomato^+^ cells.

### Library preparation and sequencing

Single-cell suspensions with an average cell viability of 80% were loaded onto the 10× Genomics Chromium Platform (10X Genomics, Pleasanton, CA, USA) to recover 6,000 single-cell-containing gel beads in emulsion (GEMs). Single Cell RNA-seq libraries were prepared according to 10x Genomics manufacturing protocols (Chromium Single Cell 3ʹ GEM, Library & Gel Bead Kit v3, 16 rxns PN-1000075. User Guide CG000183 Rev A) using a Veriti thermal cycler (ThermoFisher Scientific, Waltham, MA, USA). cDNA and Library quality were assessed using an Agilent Bioanalyzer 2100 (Agilent, Santa Clara, CA, USA). Quantification of Library was performed using StepOne Real-Time PCR System (ThermoFisher Scientific, Waltham, MA, USA). Sequencing was done on the Illumina Hiseq 2500 paired-end sequencing 29 + 8 + 101 bp. All developmental samples were sequenced as 2 lanes per sample on the Hiseq 2500 Rapid flowcell, and the mouse tumour samples were sequenced on the Hiseq 2500 High-throughput flowcell as 2 lanes per sample or 8 lanes for a pool of 5 samples.

### Magnetic resonance imaging

All MR imaging was performed at the STTARR Facility (www.sttarr.ca), using a 7 Tesla preclinical system (Biospec 70/30 USR, Bruker Corporation, Ettlingen, DE), equipped with the B-GA12 gradient coil insert, 7.2 cm inner diameter RF transmit coil, plus dedicated murine brain RF receiver coil and its associated slider bed. Anaesthetized mice (1.8% isoflurane in oxygen) were positioned on the slider bed in prone position. A respiratory pillow was fixed anteriorly to the abdomen of each mouse, for monitoring of respiratory rate during imaging (SA Instruments, Inc., Stony Brook, NY). Imaging consisted of high resolution multi-slice 2D T2-weighted imaging, with full brain coverage in its vertical/axial plane (RARE technique, echo time 72 ms; repetition time 4 s, RARE factor 16; 160 × 160 image pixels over a 16 × 16 mm field-of-view for 0.1 mm in-plane resolution; 25 slices; 0.5 mm slice thickness; 4 averages; 5 min 12 s acquisition time). Tumours were visualized using MIPAV software (National Institutes of Health, Bethesda, MD) as dicom image viewer.

### Tissue processing for immunostaining and histology

All samples were wax embedded and processed as previously reported^69^. The following antibodies and dilutions were used: Anti-Sox2 (R&D cat# af2018) at 1:150, Anti-Nestin (Novus cat# NB100-1604) at 1:500 and Anti-pdgfra (R&D cat# af1062) at 1:20, Anti-Goat IgG (H + L), made in horse, biotinylated (Vector labs cat# BA-9500) at 1:200 and Anti-chicken IgG (H + L), made in goat, biotinylated (Vector labs cat#BA-9010) at 1:200 dilution.

### Single-cell RNA-seq data processing

The raw single-cell RNA sequencing fastq files were aligned and quantified using Cell Ranger (v3.1.0, 10× Genomics Inc.) and the mm10 mouse reference (Cell Ranger reference, v3.00). Cells with fewer than 1000 genes detected were considered to be empty droplets or nuclei and removed from the data-set. For doublet detection, the over-clustering approach by Pijuan-Sala et al. in combination with Scrublet (v0.2.1) was adapted^70,71^. In addition, a mitochondrial threshold to exclude cells with high mitochondrial content which might indicate stressed or lysing cells was determined at a per sample basis^71^. To remove any sex specific effects, the genes *Xist*, *Tsix*, *Eif2s3y*, *Ddx3y*, *Uty*, and *Kdm5d* were excluded from the analysis^30^. For the regular single cell analysis the default scanpy (v1.5.1) pipeline in python (v3.6.9) was used for highly variable gene selection, regression (counts), dimensionality reduction, clustering, etc^72^.

### Cell cycle classification

Cells were classified into G1, S, G2M cell cycle stages using cyclone as implemented in the scran (v1.12.1) package in R (v3.6.2)^73,74^. To distinguish G1 and G0 cell cycle phase, the cells were gated on *Top2a* or *Mki67* expression (any: log1p(CPM) > 1:= G1).

### Differential expression analysis

To determine the differentially expressed genes for each cluster (1 vs rest), the voom-limma pipeline was adapted from Soneson et al. 2015^75,76^. DE genes were ranked and filtered (<0.05) by corrected p-value. To determine DE cluster marker genes the pairwise LFCs between the cluster of interest and all other clusters was calculated for all DE genes and the genes were subsequently ranked by the minimum LFC.

### RNA velocity inference

For the RNA velocity inference the splicing matrices were calculated using velocyto (v0.17.17)^77^. The RNA velocity graph was computed using the scVelo (v0.2.0) pipeline^78^.

### URD lineage tree inference

The lineage tree of the neuronal and glial subset was created using URD (v1.1.1)^31^. Due to the computational limitations of the method the data-set was downsampled to 30,000 cells. As the root of the tree, the early embryonic RG cluster was picked. The tips of the tree branches were picked by subclustering the relevant cell populations and selecting the most appropriate cluster.

### SCENIC transcription factor inference

The SCENIC transcription factor inference was performed using pySCENIC (v0.10.2) following the recommended pipeline steps^79^.

### Pathway analysis

Gene lists for the pathways were retrieved from the KEGG^80^ (KEGGREST, v1.30.1) and Panther^81^ (PANTHER.db, v1.0.4) pathway databases. AUCell (v1.10.0)^82^ was then used to determine the activity of each pathway at the single cell level.

### Schematics

Schematics were prepared using Biorender (https://biorender.com).

### Clustering analysis used for the deconvolution

Quality control and clustering analysis of the scRNA-seq filtered expression matrices were performed via Seurat v3 pipeline^83,84^. Low-quality cells for each dataset were defined stringently as cells with less than 1000 genes expressed, mitochondrial gene content above 5-15%, potential doublets as cells exhibiting aberrantly high gene counts (4-5 S.D.s above median). The expression matrices of all samples were aggregated and normalized together using Seurat’s global scaling normalization method. Highly variable genes across the datasets were identified using Seurat’s ‘vst’ method. Cells were then clustered using the SNN method after PCA analysis, and Louvain algorithm for modularity optimization. Clusters were then visualized using Uniform Manifold Approximation and Projection (UMAP). The mouse tdTomato^+^ tumour replicates were normalized and clustered together with the normal P111 GFP^+^ sample via Seurat. Normal cells from the tumour replicates were distinguished from tumour cells based on their clustering with the normal cells of the P111 GFP^+^ dataset. The identified tumour clusters from each tumour replicate were clustered separately, with the optimal resolution chosen based on how statistically significant the difference is between the differentially expressed genes of the clusters. Integration/co-clustering of the mouse tumour and developmental atlas cells was performed using Harmony^85^.

### Clustering analysis of scRNA-seq human tumour data

The IDH-wild type GBM dataset was collected from Neftel et al., *Cell 2019*. The normalized TPM expression matrices (7,930 cells by Smart-seq2) were obtained from the Gene Expression Omnibus (GEO) (GSE131928), analyzed, and clustered via Seurat v3. The patients were clustered aggregately and individually. For the individual patient clustering, the average expression was calculated for each cluster, and used as a mixture input for the deconvolution analysis, described below. The other GBM scRNA-seq dataset was collected from Richards et al., *Nature Cancer 2021*, the normal brain and immune cells were excluded based on the InferCNV analysis and immune markers expression prior to the individual sample clustering analysis. The human tumour GSCs scRNA-seq dataset was also collected from Richards et al., *Nature Cancer 2021*. Integration/co-clustering of the human GSCs with the normal mouse cells from the developmental atlas was performed using Harmony after orthologue mapping. scRNA-seq IDH1-mutant astrocytoma tumour samples were collected from Venteicher et al., *Science 2017*. Processed and normalized expression data (GSE89567) of 6341 cells from 10 tumours sequenced by Smart-seq2 were collected, analyzed, and clustered via Seurat v3. Individual patient clusters were used in the deconvolution analysis as above. Smart-seq2 single-cell oligodendroglioma tumours were obtained from Tirosh et al., *Science 2016*. Processed matrices of ~1000 cells from 6 patients (GSE70630) were obtained and analyzed in the same method described above. Here, the aggregated clustering was used for deconvolution analysis. Supratentorial paediatric ependymomas were obtained from the Michael Taylor Lab at SickKids (who deposited the data in the European Genome-phenome Archive (EGA) under accession number EGAS00001006237). The raw data was processed using the cellranger pipeline, and the filtered expression matrices were analyzed and clustered aggregately using Seurat v3. Tumour clusters were distinguished from non-tumour clusters through differential gene expression.

### Cell-type deconvolution analysis of scRNA-seq tumour data

To construct a signature gene expression matrix from the mouse developmental scRNA-seq dataset, differential gene expression was performed between all the clusters using Wilcoxon rank sum test method, with a pct cut-off of 0.5 and average log fold-change cut-off of 0.5. In order to generate reliable input expression profiles, unknown clusters with very low number of cells were discarded from the analysis. The average normalized expression of each cluster was calculated, and a signature matrix was created using the differentially expressed genes. Mouse genes involved in cell cycling/proliferation, ribosome biogenesis, mitochondrial and apoptosis-related genes were obtained from Ensembl’s biomart^86^ and removed. Human orthologues of mouse genes were identified from Ensembl’s biomart, and used to create the final signature gene expression matrix. CIBERSORT/CIBERSORTx^45,46^, DWLS (weighted least squares approach)^87^, BisqueRNA (regression-based deconvolution and marker-based decomposition)^88^ were examined to deconvolute human scRNA-seq normal foetal cell types from the mouse cell types. CIBERSORT/CIBERSORTx was chosen based on the best correlation with human cell types and consistency across different datasets and varying signature matrices. The normalized average expression for each cluster was used as the mixture for scRNA-seq tumour datasets. Hierarchical clustering (via pheatmap package) of patient samples/clusters based on the calculated relative fraction values from CIBERSORT/CIBERSORTx was then performed. A matrix layout intersection was produced to show the associations and overlap between the patient clusters, using relative abundance of 0.2 as cutoff for bulk scRNA-seq^89^. Shared gene lists were obtained by calculating the difference in average normalized expression between the tumor and normal cells from the signature matrix.

The following R packages were used for the cancer matching analysis: Seurat v3.1.4, biomaRt v2.42.1, pheatmap v1.0.12, ggplot2 v3.3.2, UpsetR v1.4.0, BisqueRNA v1.0.4, CIBSERSORT (https://cibersort.stanford.edu), CIBERSORTx (https://cibersortx.stanford.edu).

### Reporting summary

Further information on research design is available in the Nature Research Reporting Summary linked to this article.

## Supplementary information

Supplementary Information

Description of Additional Supplementary Files

Supplementary Data 1

Supplementary Data 2

Reporting Summary

## Data Availability

The raw and processed scRNA-seq data of the mouse developmental atlas and the mouse tumour samples generated in this study have been deposited in GEO under accession number (GSE200202). The human IDH-WT GBM^1,51^, IDH-mutant GBM^4^, oligodendroglioma^3^, GSC lines^51^ and human foetal^50^ scRNA-seq datasets were obtained from previous publications and are publicly available datasets in GEO, EGA and the database of Genotypes and Phenotypes (dbGaP) under the following accession numbers (GSE131928, GSE89567, GSE70630, EGAS00001004656, phs001836). The human supratentorial ependymoma scRNA-seq data was obtained from the Michael Taylor Lab at SickKids (who deposited the data in EGA under accession number (EGAS00001006237)). The remaining data are available within the Article, Supplementary Information or Source Data file. Source data are provided with this paper.

## Source data

Source Data

## Code availability

The code used for the mouse developmental atlas analysis can be retrieved here: https://github.com/BenSimonsLab/Hamed_Nat-Commun_2022.
