# Supplementary material for: A brain precursor atlas reveals the acquisition of developmental-like states in adult cerebral tumours
Source: Nat Commun. 2022 Jul 19;13:4178. doi: 10.1038/s41467-022-31408-y (PMC9296666; doi:10.1038/s41467-022-31408-y)
Supplement: Supplementary file 1 — Supplementary Information [file 41467_2022_31408_MOESM1_ESM.pdf]

a

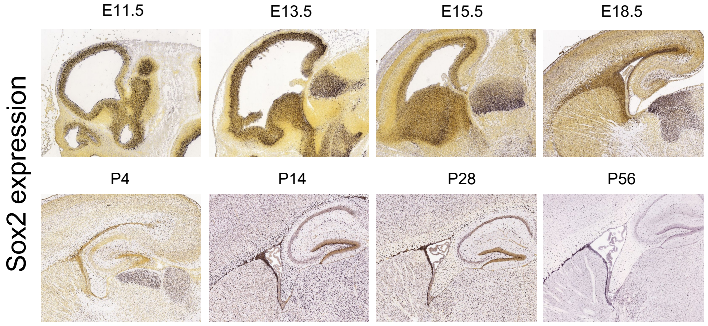
E11.5
E13.5
E15.5
E18.5
Sox2 expression
P4
P14
P28
P56

b

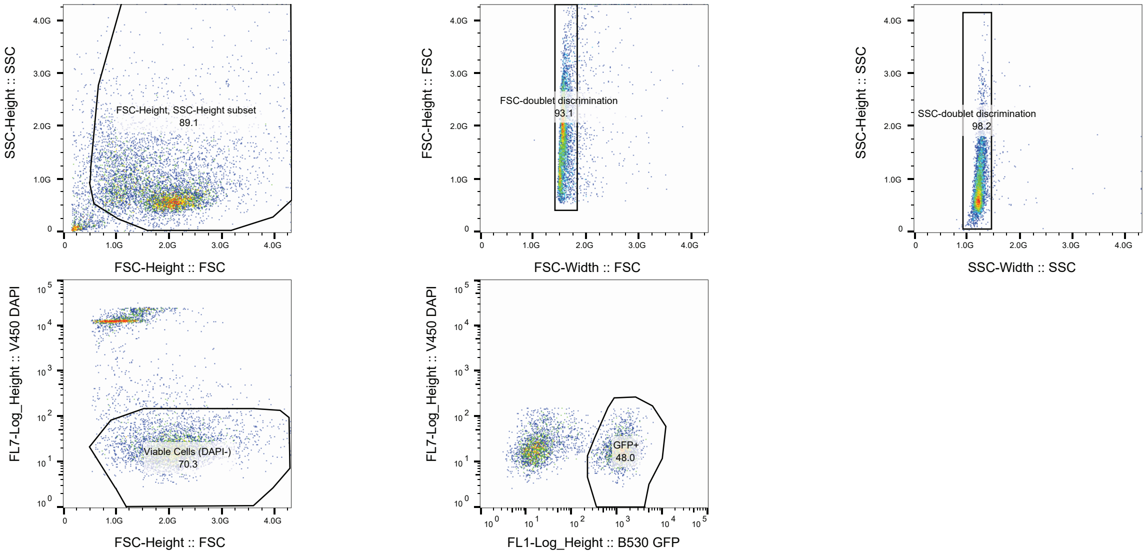
SSC-Height :: SSC
FSC-Height, SSC-Height subset
89.1
FSC-Height :: FSC
FSC-Height :: FSC
FSC-doublet discrimination
93.1
FSC-Width :: FSC
SSC-Height :: SSC
SSC-doublet discrimination
98.2
SSC-Width :: SSC
FL7-Log_Height :: V450 DAPI
Viable Cells (DAPI-)
70.3
FSC-Height :: FSC
FL7-Log_Height :: V450 DAPI
GFP+
48.0
FL1-Log_Height :: B530 GFP

c

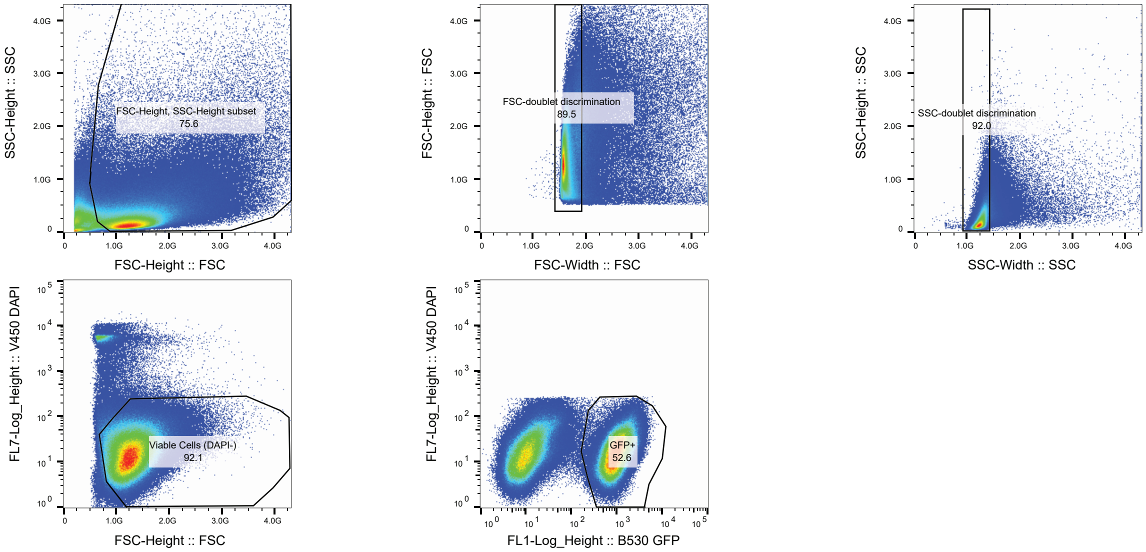
SSC-Height :: SSC
FSC-Height, SSC-Height subset
75.6
FSC-Height :: FSC
FSC-Height :: FSC
FSC-doublet discrimination
89.5
FSC-Width :: FSC
SSC-Height :: SSC
SSC-doublet discrimination
92.0
SSC-Width :: SSC
FL7-Log_Height :: V450 DAPI
Viable Cells (DAPI-)
92.1
FSC-Height :: FSC
FL7-Log_Height :: V450 DAPI
GFP+
52.6
FL1-Log_Height :: B530 GFP

Supplementary Fig. 1 | The expression of Sox2 across cerebral development and the FACS gating strategy. a, In-situ hybridization staining of sagittal embryo/adult brain slices showing the expression of Sox2 across 8 developmental timepoints. In-situ hybridization images were obtained from the Allen Brain Map: Developing Mouse Brain Atlas. **b, c,** FACS plots of the collection strategy of the $GFP^{+/-}$ cells from embryonic day E12.5 (b) and postnatal day 2 (c) *Sox2eGFP* mouse brain samples.

a

Timepoint	QC pass [%]	n cells post QC [GFP+ (Sox2)]	n cells post QC [GFP−]	n cells post QC [total]
E12.5	78.31	6188	4760	10948
E14.5	80.69	4533	4157	8690
E16.5	52.90	5847	2761	8608
P0	57.44	4908	4737	9645
P2	67.11	5019	5331	10350
P7	66.41	6294	7688	13982
P13	71.51	1762	1263	3025
P19	67.23	4558	3785	8343
P39	77.47	8008	4208	12216
P111	85.87	4351	4439	8790
P365	83.79	4461	3461	7922

b

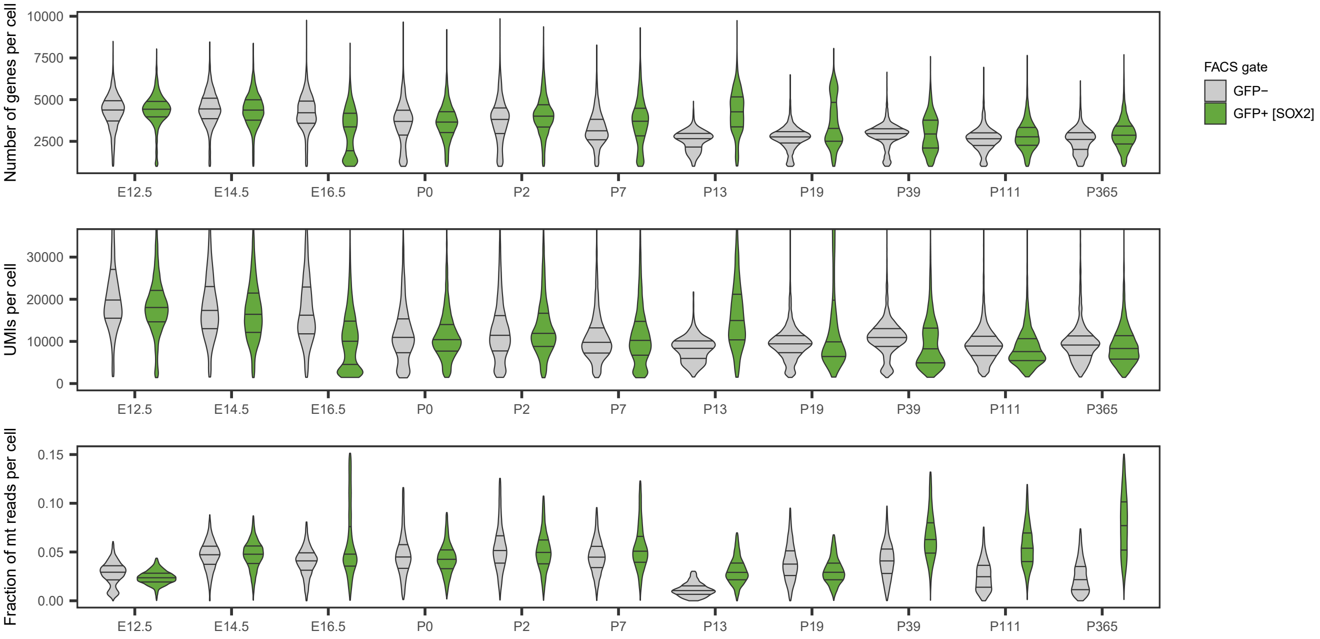

c

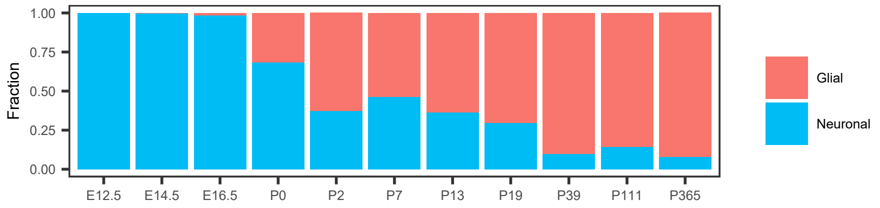

d

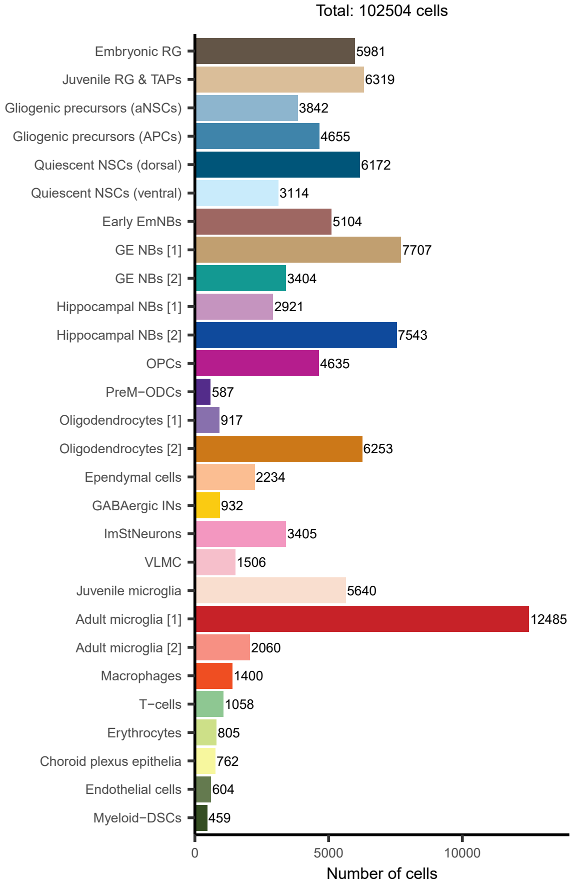

e

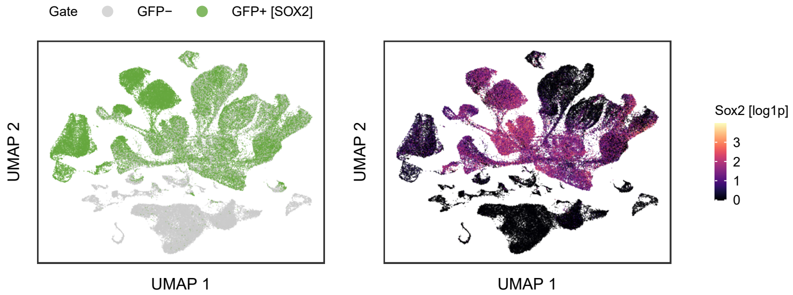

Supplementary Fig. 2 | Quality control and the Sox2/GFP analysis. a, Table summarizing some of the QC statistics for each timepoint in the mouse atlas. **b,** Violin plots of the number of genes/cell, UMIs/cell and fraction of mitochondrial reads/cell across the different samples. Colour bars indicate the sample type based on the FACS gating. **c,** Bar plot showing the normalized relative fraction of the Neuronal and Glial cell types grouped by timepoint. Colours correspond to the 2 cell types. The annotation is based on logistic regression from Zeisel et al., 2018 (see Fig. 1d). **d,** Bar plot showing the number of cells identified for each cell type in the dataset (see Fig. 1b). **e,** Left: two-dimensional UMAP visualization of the FACS GFP^+/GFP^- gating. Shown on the right is the log1p-expression of Sox2 overlaid on the UMAP plot of the single cells collected from the various mouse brain samples. Each dot represents a single cell and the colour scale reflects the gene expression level.
Source data are provided as a Source Data file. Abbreviations as in Fig. 1.

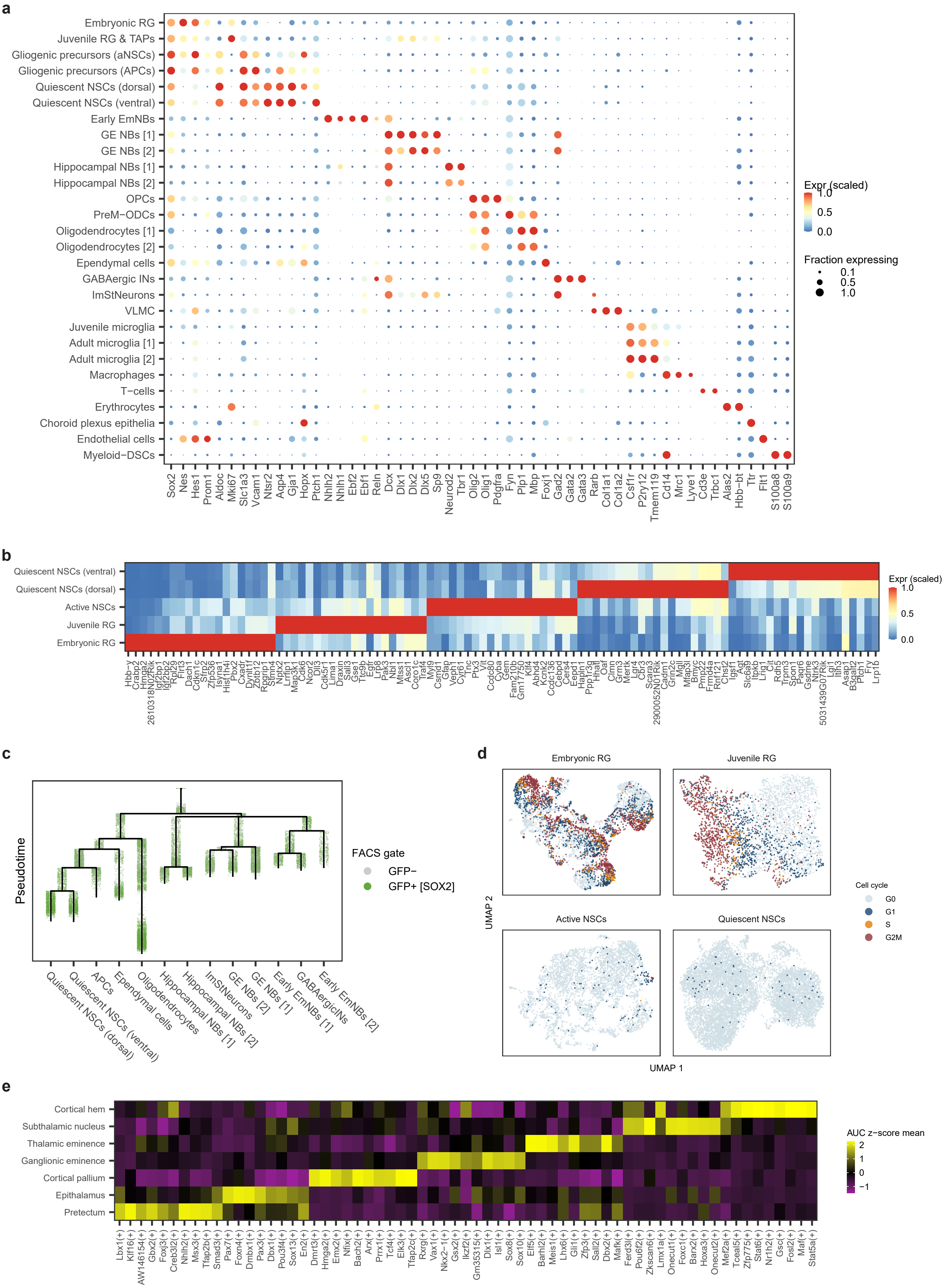

a
Embryonic RG
Juvenile RG & TAPs
Gliogenic precursors (aNSCs)
Gliogenic precursors (APCs)
Quiescent NSCs (dorsal)
Quiescent NSCs (ventral)
Early EmNBs
GE NBs [1]
GE NBs [2]
Hippocampal NBs [1]
Hippocampal NBs [2]
OPCs
PreM−ODCs
Oligodendrocytes [1]
Oligodendrocytes [2]
Ependymal cells
GABAergic INs
ImStNeurons
VLMC
Juvenile microglia
Adult microglia [1]
Adult microglia [2]
Macrophages
T−cells
Erythrocytes
Choroid plexus epithelia
Endothelial cells
Myeloid−DSCs
Sox2 Nes Hes1 Prom1 Aldoc Mki67 Slc1a3 Vcam1 Ntsr2 Aqp4 Gja1 Hopx Ptch1 Nhlh2 Nhlh1 Ebf2 Ebf1 Reln Dcx Dlx1 Dlx2 Dlx5 Sp9 Neurod2 Tbr1 Olig2 Olig1 Pdgfra Fyn Plp1 Mbp Foxj1 Gad2 Gata2 Gata3 Rarb Col1a1 Col1a2 Csf1r P2ry12 Tmem119 Cd14 Mrc1 Lyve1 Cd3e Trbc1 Alas2 Hbb−bt Ttr Flt1 S100a8 S100a9
Expr (scaled)
1.0
0.5
0.0
Fraction expressing
0.1
0.5
1.0
b
Quiescent NSCs (ventral)
Quiescent NSCs (dorsal)
Active NSCs
Juvenile RG
Embryonic RG
Expr (scaled)
1.0
0.5
0.0
c
Pseudotime
FACS gate
GFP−
GFP+ [SOX2]
Quiescent NSCs (dorsal)
Quiescent NSCs (ventral)
APCs
Ependymal cells
Oligodendrocytes
Hippocampal NBs [1]
Hippocampal NBs [2]
ImStNeurons
GE NBs [2]
GE NBs [1]
Early EmNBs [1]
GABAergicINs
Early EmNBs [2]
d
Embryonic RG
Juvenile RG
Active NSCs
Quiescent NSCs
UMAP 2
UMAP 1
Cell cycle
G0
G1
S
G2M
e
Cortical hem
Subthalamic nucleus
Thalamic eminence
Ganglionic eminence
Cortical pallium
Epithalamus
Pretectum
Lbx1(+) Klf16(+) AW146154(+) Gbx2(+) Foxj3(+) Creb3l2(+) Nhlh2(+) Msx3(+) Tfap2b(+) Smad3(+) Pax7(+) Foxn4(+) Dmbx1(+) Pax3(+) Dbx1(+) Pou3f4(+) Sox13(+) En2(+) Dmrt3(+) Hmga2(+) Emx2(+) Nfix(+) Bach2(+) Arx(+) Prrx1(+) Tcf4(+) Elk3(+) Tfap2c(+) Rxrg(+) Vax1(+) Nkx2−1(+) Gsx2(+) Ikzf2(+) Gm35315(+) Dlx1(+) Isl1(+) Sox8(+) Sox10(+) Elf5(+) Barhl2(+) Meis1(+) Lhx6(+) Gli1(+) Zfp3(+) Sall2(+) Dbx2(+) Mafk(+) Ferd3l(+) Pou6f2(+) Zkscan6(+) Lmx1a(+) Onecut1(+) Foxc1(+) Barx2(+) Hoxa3(+) Onecut2(+) Mef2a(+) Tceal5(+) Zfp775(+) Stat6(+) Nr1h2(+) Gsc(+) Fosl2(+) Maf(+) Stat5a(+)
AUC z-score mean
2
1
0
−1

Supplementary Fig. 3 | a, Dot plot showing the normalized expression levels of lineage-specific marker genes used to annotate the clusters in Fig. 1b. The colour scale reflects the gene expression level and the dot size indicates the percentage of cells in each cluster expressing that gene. **b,** Heatmap of the top 20 DE marker genes identified per cluster from analyzing the clusters in Fig. 2a. Colour scale indicates the scaled mean expression in each cluster. **c,** Visualization of the FACS GFP^+/GFP^- gating overlaid on the lineage tree of the neuronal/glial cells (see Fig. 2e). Each dot represents a single cell and the colour scale reflects the FACs gating GFP^+/GFP^-. **d,** UMAP plot of each of the four precursor populations identified in Fig. 2a with the cell cycle stages indicated by the colours. Each dot represents a single cell. Cycling stages were identified based on Cyclone (see Methods). The scale colours correspond to the cell cycle stages. **e,** Heatmap generated from the SCENIC analysis of the embryonic RG subtypes identified in Fig. 3a showing the top 10 TF regulons per cluster. Colour scale indicates the mean regulon score. Abbreviations as in Fig. 1.

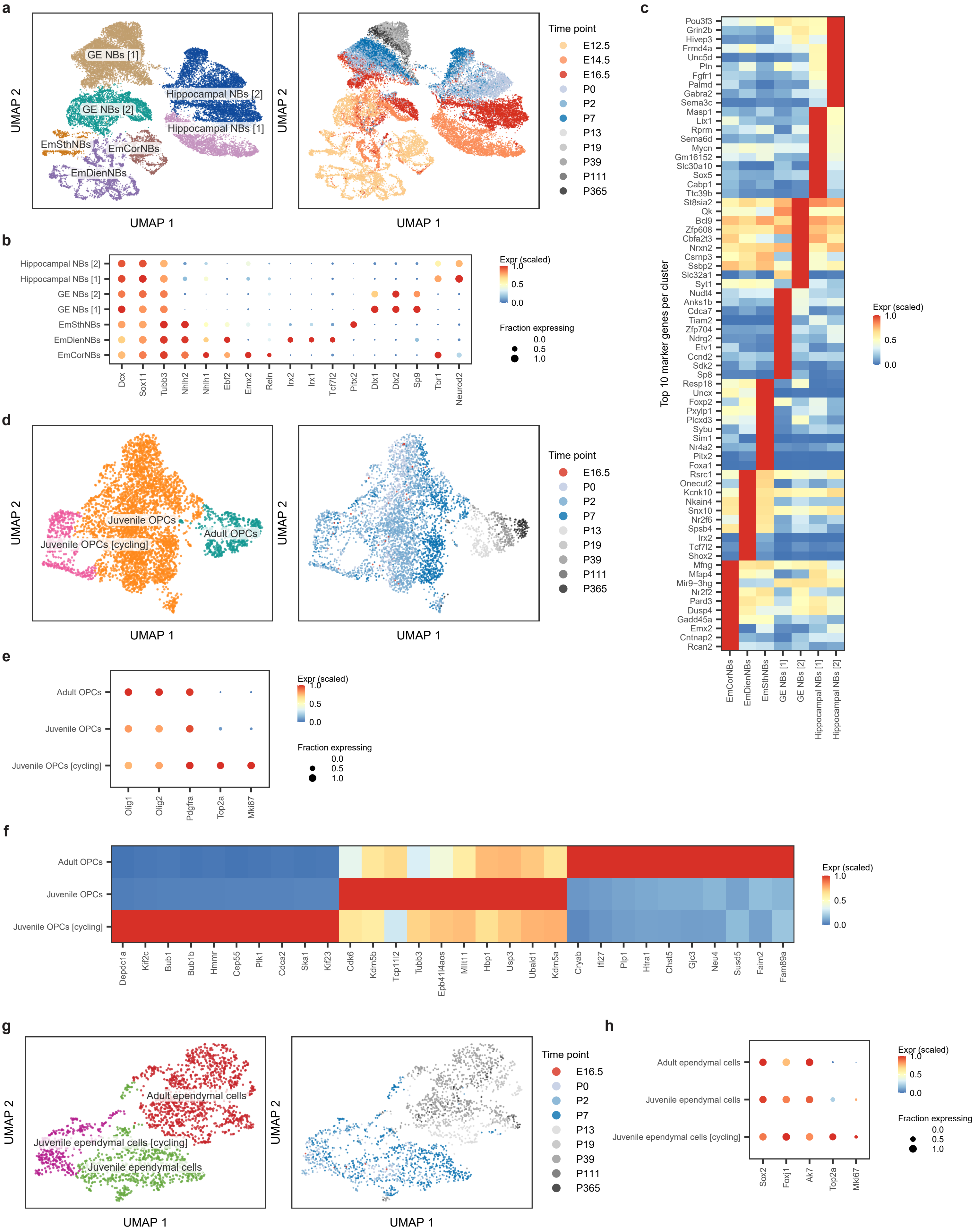
a
GE NBs [1]
GE NBs [2]
Hippocampal NBs [2]
Hippocampal NBs [1]
EmSthNBs
EmCorNBs
EmDienNBs
UMAP 2
UMAP 1
Time point
E12.5
E14.5
E16.5
P0
P2
P7
P13
P19
P39
P111
P365
b
Hippocampal NBs [2]
Hippocampal NBs [1]
GE NBs [2]
GE NBs [1]
EmSthNBs
EmDienNBs
EmCorNBs
Dcx
Sox11
Tubb3
Nhlh2
Nhlh1
Ebf2
Emx2
Reln
Irx2
Irx1
Tcf7l2
Pitx2
Dlx1
Dlx2
Sp9
Tbr1
Neurod2
Expr (scaled)
1.0
0.5
0.0
Fraction expressing
0.0
0.5
1.0
c
Top 10 marker genes per cluster
Pou3f3
Grin2b
Hivep3
Frmd4a
Unc5d
Ptn
Fgfr1
Palmd
Gabra2
Sema3c
Masp1
Lix1
Rprm
Sema6d
Mycn
Gm16152
Slc30a10
Sox5
Cabp1
Ttc39b
St8sia2
Qk
Bcl9
Zfp608
Cbfa2t3
Nrxn2
Csrnp3
Ssbp2
Slc32a1
Syt1
Nudt4
Anks1b
Cdca7
Tiam2
Zfp704
Ndrg2
Etv1
Ccnd2
Sdk2
Sp8
Resp18
Uncx
Foxp2
Pxylp1
Plcxd3
Sybu
Sim1
Nr4a2
Pitx2
Foxa1
Rsrc1
Onecut2
Kcnk10
Nkain4
Snx10
Nr2f6
Spsb4
Irx2
Tcf7l2
Shox2
Mfng
Mfap4
Mir9−3hg
Nr2f2
Pard3
Dusp4
Gadd45a
Emx2
Cntnap2
Rcan2
EmCorNBs
EmDienNBs
EmSthNBs
GE NBs [1]
GE NBs [2]
Hippocampal NBs [1]
Hippocampal NBs [2]
Expr (scaled)
1.0
0.5
0.0
d
Juvenile OPCs
Juvenile OPCs [cycling]
Adult OPCs
UMAP 2
UMAP 1
Time point
E16.5
P0
P2
P7
P13
P19
P39
P111
P365
e
Adult OPCs
Juvenile OPCs
Juvenile OPCs [cycling]
Olig1
Olig2
Pdgfra
Top2a
Mki67
Expr (scaled)
1.0
0.5
0.0
Fraction expressing
0.0
0.5
1.0
f
Adult OPCs
Juvenile OPCs
Juvenile OPCs [cycling]
Depdc1a
Kif2c
Bub1
Bub1b
Hmmr
Cep55
Plk1
Cdca2
Ska1
Kif23
Cdk6
Kdm5b
Tcp11l2
Tubb3
Epb41l4aos
Mllt11
Hbp1
Usp3
Ubald1
Kdm5a
Cryab
Ifi27
Plp1
Htra1
Chst5
Gjc3
Neu4
Susd5
Faim2
Fam89a
Expr (scaled)
1.0
0.5
0.0
g
Adult ependymal cells
Juvenile ependymal cells [cycling]
Juvenile ependymal cells
UMAP 2
UMAP 1
Time point
E16.5
P0
P2
P7
P13
P19
P39
P111
P365
h
Adult ependymal cells
Juvenile ependymal cells
Juvenile ependymal cells [cycling]
Sox2
Foxj1
Ak7
Top2a
Mki67
Expr (scaled)
1.0
0.5
0.0
Fraction expressing
0.0
0.5
1.0

Supplementary Fig. 4 | Temporal diversity of the lineage cell-types throughout forebrain development. a, Left: two-dimensional UMAP visualization of the neuroblast clusters identified in the dataset from Fig. 1b. Each dot represents a single cell and the colours correspond to the distinct neuroblast subtypes. Right, UMAP plot of the cells coloured by timepoint. Each dot represents a single cell and the colours correspond to the different timepoints. **b,** Dot plot of the main marker genes used to differentiate/annotate the distinct neuroblast clusters. Dot size indicates the percentage of cells in each cluster expressing that gene. **c,** Heatmap of the top 10 DE marker genes identified per cluster from analyzing the clusters in (a). Colour scale indicates the scaled mean expression in each cluster. **d,** Left: two-dimensional UMAP visualization of the distinct OPC clusters identified in the dataset. Each dot represents a single cell and the colours correspond to the distinct clusters. Right: UMAP plot of the cells coloured by timepoint. Each dot represents a single cell and the colours correspond to the different timepoints. **e,** Dot plot of the main marker genes used to differentiate/annotate the distinct OPC clusters. Dot size indicates the percentage of cells in each cluster expressing that gene. **f,** Heatmap of the top 10 DE marker genes identified per cluster from analyzing the clusters (d). Colour scale indicates the scaled mean expression in each cluster. **g,** Left: two-dimensional UMAP visualization of ependymal cell clusters identified in the dataset. Each dot represents a single cell and the colours correspond to the distinct clusters. Right: UMAP plot of the cells coloured by timepoint. Each dot represents a single cell and the colours correspond to the different timepoints. **h,** Dot plot of the main marker genes used to identify ependymal cells in the dataset. Dot size indicates the percentage of cells in each cluster expressing that gene.

Abbreviations: EmThNBs: embryonic thalamic neuroblasts, EmCorNBs: embryonic cortical neuroblasts, EmSthNBs: embryonic subthalamic neuroblasts, EmDienNBs: embryonic diencephalon neuroblasts. Other abbreviations are as in Fig. 1.

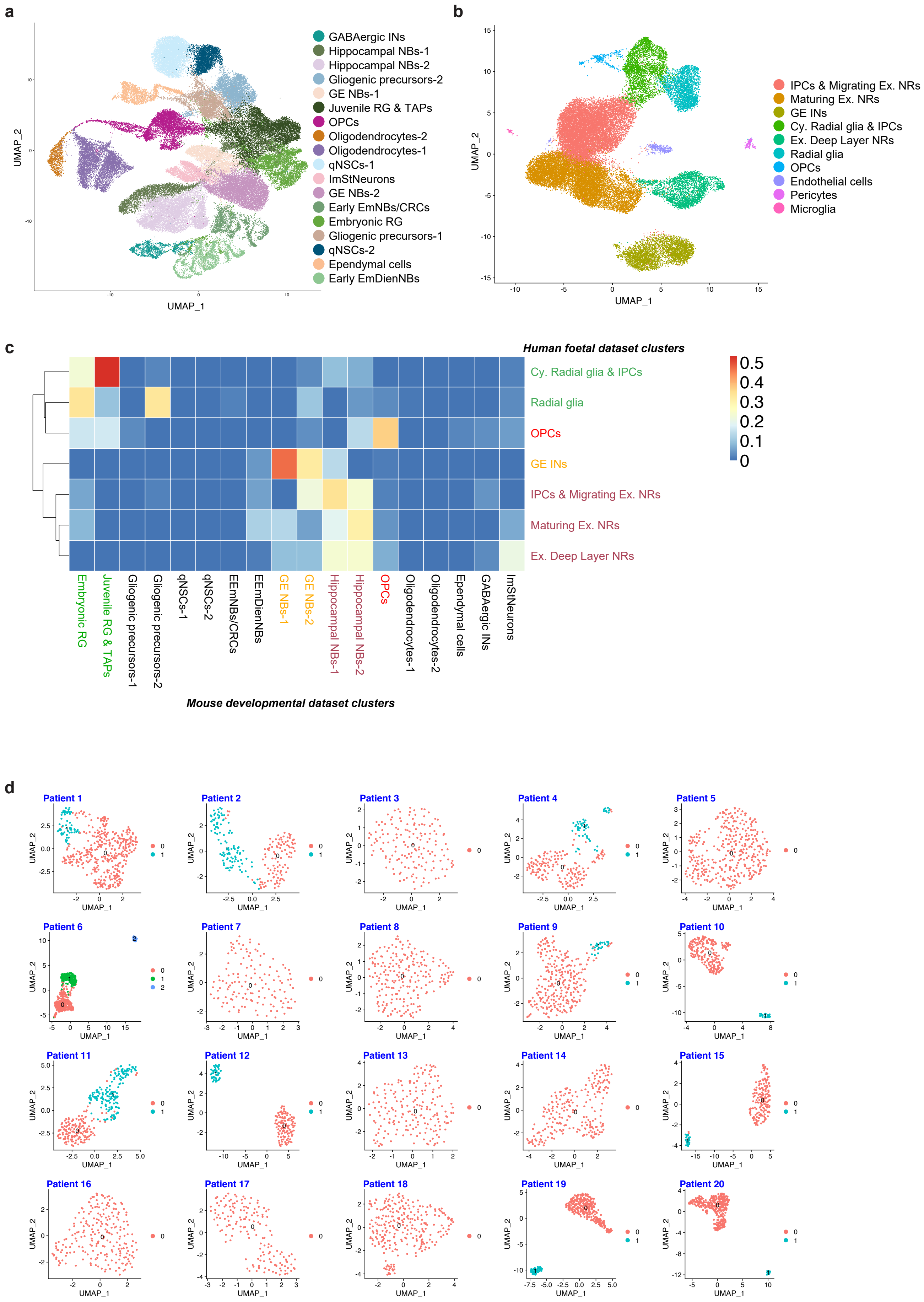
a
UMAP_1
UMAP_2
GABAergic INs
Hippocampal NBs-1
Hippocampal NBs-2
Gliogenic precursors-2
GE NBs-1
Juvenile RG & TAPs
OPCs
Oligodendrocytes-2
Oligodendrocytes-1
qNSCs-1
ImStNeurons
GE NBs-2
Early EmNBs/CRCs
Embryonic RG
Gliogenic precursors-1
qNSCs-2
Ependymal cells
Early EmDienNBs
b
UMAP_1
UMAP_2
IPCs & Migrating Ex. NRs
Maturing Ex. NRs
GE INs
Cy. Radial glia & IPCs
Ex. Deep Layer NRs
Radial glia
OPCs
Endothelial cells
Pericytes
Microglia
c
Human foetal dataset clusters
Cy. Radial glia & IPCs
Radial glia
OPCs
GE INs
IPCs & Migrating Ex. NRs
Maturing Ex. NRs
Ex. Deep Layer NRs
0.5
0.4
0.3
0.2
0.1
0
Embryonic RG
Juvenile RG & TAPs
Gliogenic precursors-1
Gliogenic precursors-2
qNSCs-1
qNSCs-2
EEmNBs/CRCs
EEmDienNBs
GE NBs-1
GE NBs-2
Hippocampal NBs-1
Hippocampal NBs-2
OPCs
Oligodendrocytes-1
Oligodendrocytes-2
Ependymal cells
GABAergic INs
ImStNeurons
Mouse developmental dataset clusters
d
Patient 1
Patient 2
Patient 3
Patient 4
Patient 5
Patient 6
Patient 7
Patient 8
Patient 9
Patient 10
Patient 11
Patient 12
Patient 13
Patient 14
Patient 15
Patient 16
Patient 17
Patient 18
Patient 19
Patient 20
UMAP_1
UMAP_2

Supplementary Fig. 5 | Mapping human foetal scRNA-seq data to the mouse atlas reveals high similarities between the human foetal RG and the mouse embryonic and juvenile RG. a, Two-dimensional UMAP visualization of the clustered mouse atlas (after excluding the immune and vascular clusters) showing the neuronal and glial lineages which were used for the deconvolution analysis. Each dot represents a single cell and the colours correspond to the different clusters. **b,** Two-dimensional UMAP visualization of the human foetal dataset from Polioudakis et al., 2019 coloured by cell type. Each dot represents a single cell. **c,** Aligning the mouse atlas neuronal and glial clusters (x-axis) to the neuronal and glial cell types identified in the human foetal brain scRNA-seq data (y-axis) showing relative fraction of the mouse cell-types for each human cluster. The coloured cluster names indicate matching clusters between the mouse and human datasets. **d,** Clustering analysis of the scRNA-seq data from each patient from the Smart-seq2 adult GBM dataset (from Neftel et al., 2019), which was used in the deconvolution analysis in Fig. 4a (see Methods and Supplementary Data 1). Each dot represents a single cell and colours correspond to the distinct clusters.
Abbreviations: Cy.: cycling, IPCs: intermediate progenitor cells, GE INs: ganglionic eminence interneurons, Ex. NRs: excitatory neurons. Other abbreviations are as in Fig. 4.

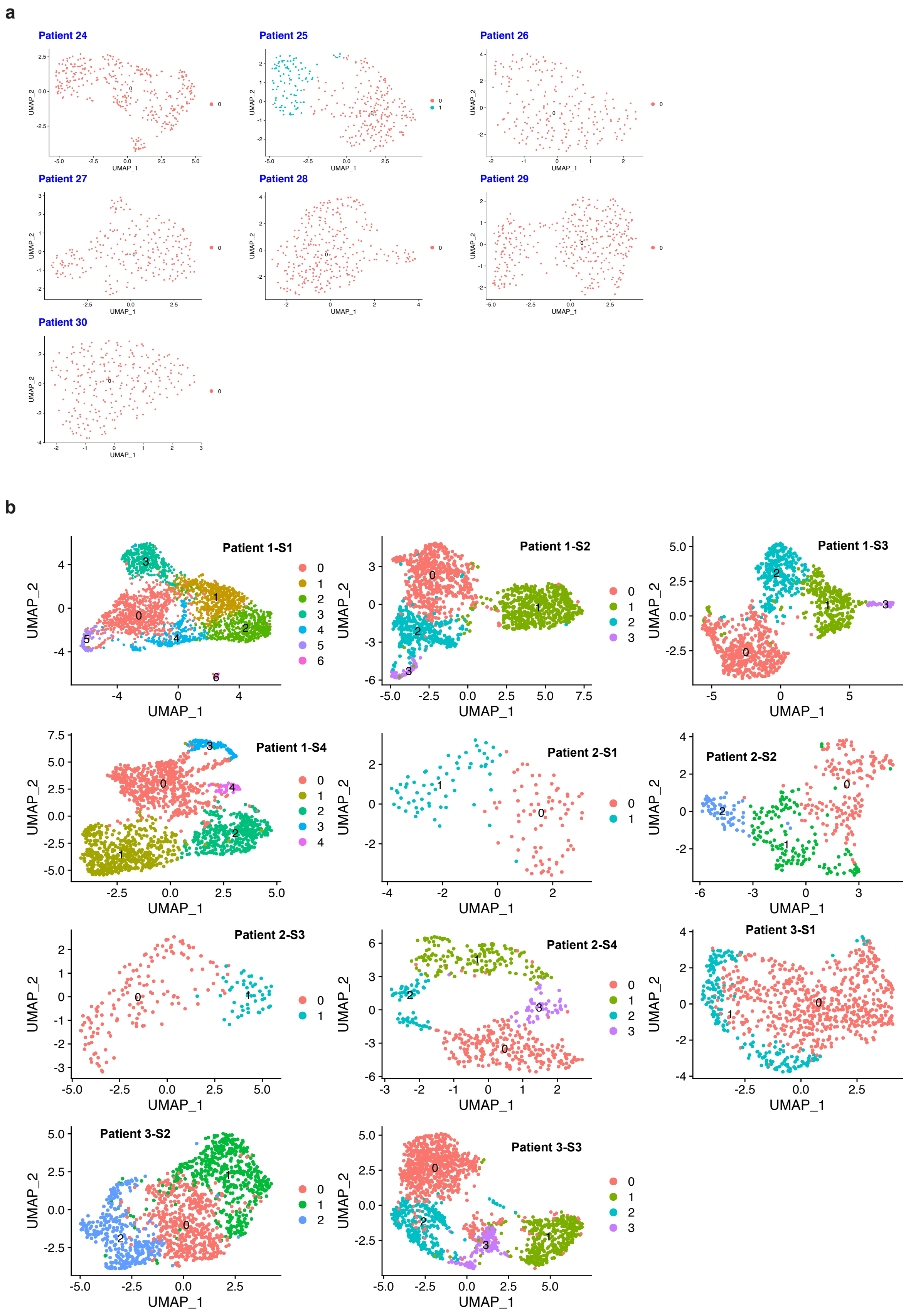
a
Patient 24
Patient 25
Patient 26
Patient 27
Patient 28
Patient 29
Patient 30
UMAP_1
UMAP_2
b
Patient 1-S1
Patient 1-S2
Patient 1-S3
Patient 1-S4
Patient 2-S1
Patient 2-S2
Patient 2-S3
Patient 2-S4
Patient 3-S1
Patient 3-S2
Patient 3-S3
UMAP_1
UMAP_2

Supplementary Fig. 6 | a, Clustering analysis of the scRNA-seq data from each patient from the Smart-seq2 paediatric GBM dataset (from Neftel et al., 2019), which was used in the deconvolution analysis in Fig. 4. (see Methods and Supplementary Data 1). **b,** Clustering analysis of the scRNA-seq data from the GBM dataset (from Richards et al., 2021), which was used in the deconvolution analysis in Supplementary Fig. 8a (see Methods and Supplementary Data 1). Each patient sample is clustered separately and the clusters are denoted as e.g. “Patient#1-Sample#1-cluster#1” = “Patient 1-S1-C1” (see Supplementary Data 1). Normal brain and immune cells were excluded prior to the clustering analysis (based on the InferCNV analysis and immune markers expression). Each dot represents a single cell and colours correspond to the distinct clusters.

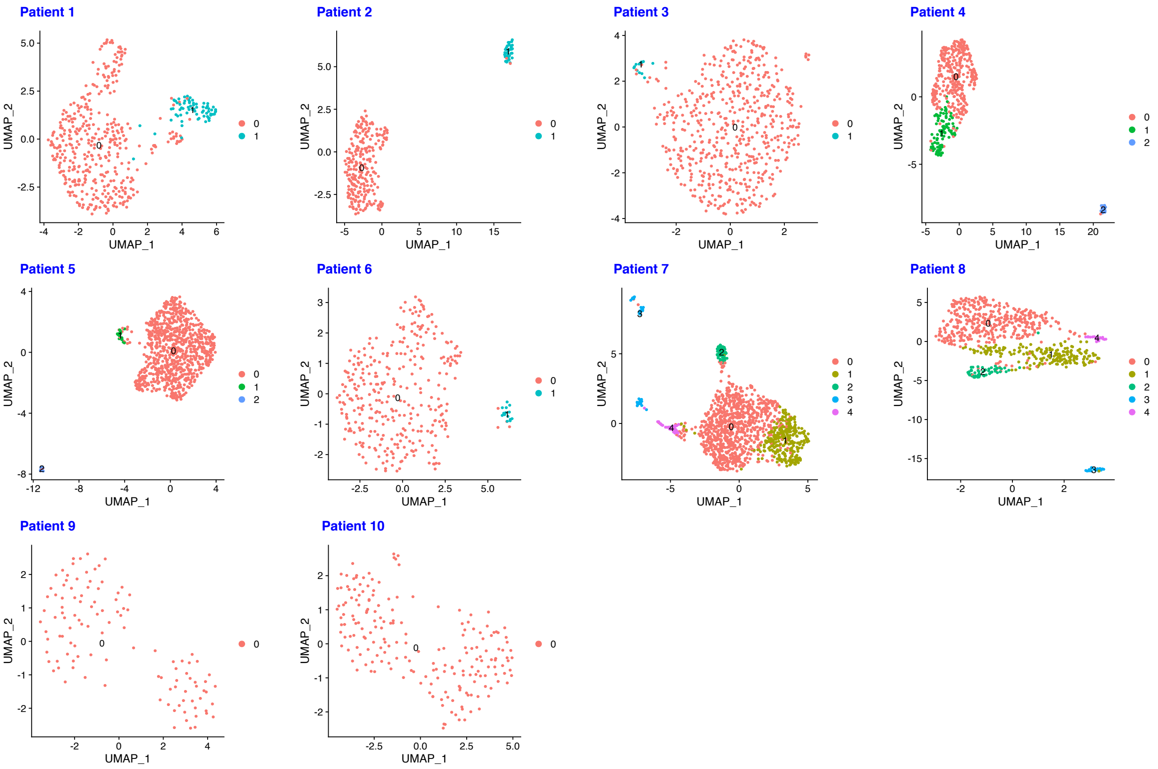
Patient 1
Patient 2
Patient 3
Patient 4
Patient 5
Patient 6
Patient 7
Patient 8
Patient 9
Patient 10
UMAP_1
UMAP_2

Supplementary Fig. 7 | Clustering analysis for each patient from the scRNA-seq of the IDH-mutant glioma dataset (Venteicher et al., 2017), which was used in the deconvolution analysis in Fig. 5a. See Methods and Supplementary Data 1.

a

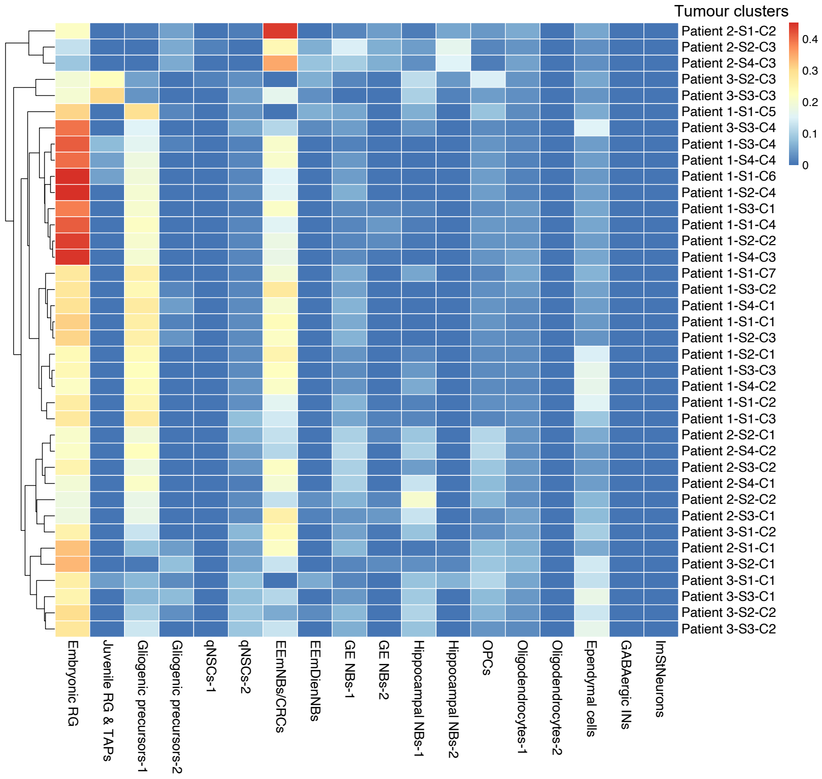
Tumour clusters
0.4
0.3
0.2
0.1
0
Patient 2-S1-C2
Patient 2-S2-C3
Patient 2-S4-C3
Patient 3-S2-C3
Patient 3-S3-C3
Patient 1-S1-C5
Patient 3-S3-C4
Patient 1-S3-C4
Patient 1-S4-C4
Patient 1-S1-C6
Patient 1-S2-C4
Patient 1-S3-C1
Patient 1-S1-C4
Patient 1-S2-C2
Patient 1-S4-C3
Patient 1-S1-C7
Patient 1-S3-C2
Patient 1-S4-C1
Patient 1-S1-C1
Patient 1-S2-C3
Patient 1-S2-C1
Patient 1-S3-C3
Patient 1-S4-C2
Patient 1-S1-C2
Patient 1-S1-C3
Patient 2-S2-C1
Patient 2-S4-C2
Patient 2-S3-C2
Patient 2-S4-C1
Patient 2-S2-C2
Patient 2-S3-C1
Patient 3-S1-C2
Patient 2-S1-C1
Patient 3-S2-C1
Patient 3-S1-C1
Patient 3-S3-C1
Patient 3-S2-C2
Patient 3-S3-C2
Embryonic RG
Juvenile RG & TAPs
Gliogenic precursors-1
Gliogenic precursors-2
qNSCs-1
qNSCs-2
EEmNBs/CRCs
EEmDienNBs
GE NBs-1
GE NBs-2
Hippocampal NBs-1
Hippocampal NBs-2
OPCs
Oligodendrocytes-1
Oligodendrocytes-2
Ependymal cells
GABAergic INs
ImStNeurons

b

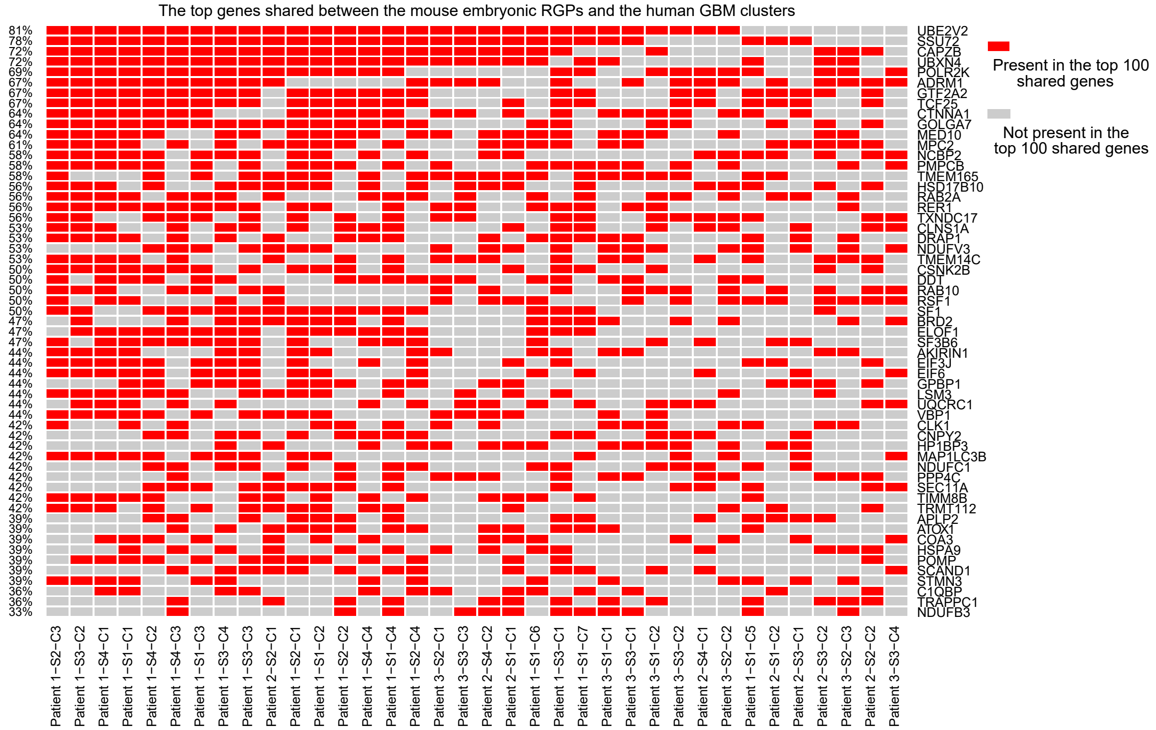
The top genes shared between the mouse embryonic RGPs and the human GBM clusters
Present in the top 100 shared genes
Not present in the top 100 shared genes
UBE2V2 81%
SSU72 78%
CAPZB 72%
UBXN4 72%
POLR2K 69%
ADRM1 67%
GTF2A2 67%
TCF25 67%
CTNNA1 64%
GOLGA7 64%
MED10 64%
MPC2 61%
NCBP2 58%
PMPCB 58%
TMEM165 58%
HSD17B10 56%
RAB2A 56%
RER1 56%
TXNDC17 56%
CLNS1A 53%
DRAP1 53%
NDUFV3 53%
TMEM14C 53%
CSNK2B 50%
DDT 50%
RAB10 50%
RSF1 50%
SF1 50%
BRD2 47%
ELOF1 47%
SF3B6 47%
AKIRIN1 44%
EIF3J 44%
EIF6 44%
GPBP1 44%
LSM3 44%
UQCRC1 44%
VBP1 44%
CLK1 42%
CNPY2 42%
HP1BP3 42%
MAP1LC3B 42%
NDUFC1 42%
PPP4C 42%
SEC11A 42%
TIMM8B 42%
TRMT112 42%
APLP2 39%
ATOX1 39%
COA3 39%
HSPA9 39%
POMP 39%
SCAND1 39%
STMN3 39%
C1QBP 36%
TRAPPC1 36%
NDUFB3 33%
Patient 1−S2−C3
Patient 1−S3−C2
Patient 1−S4−C1
Patient 1−S1−C1
Patient 1−S4−C2
Patient 1−S4−C3
Patient 1−S1−C3
Patient 1−S3−C4
Patient 1−S3−C3
Patient 2−S2−C1
Patient 1−S2−C1
Patient 1−S1−C2
Patient 1−S2−C2
Patient 1−S4−C4
Patient 1−S1−C4
Patient 1−S2−C4
Patient 3−S2−C1
Patient 3−S3−C3
Patient 2−S4−C2
Patient 2−S1−C1
Patient 1−S1−C6
Patient 1−S3−C1
Patient 1−S1−C7
Patient 3−S1−C1
Patient 3−S3−C1
Patient 3−S1−C2
Patient 3−S3−C2
Patient 2−S4−C1
Patient 3−S2−C2
Patient 1−S1−C5
Patient 2−S1−C2
Patient 2−S3−C1
Patient 2−S3−C2
Patient 3−S2−C3
Patient 2−S2−C2
Patient 3−S3−C4

Supplementary Fig. 8 | Identifying the genes shared between the GBM cells and the matching embryonic precursors. a, Deconvolution analysis heatmap of malignant clusters from 11 scRNA-seq samples obtained from 3 adult GBM patients from Richards et al., 2021 showing the estimated relative fraction of the mouse cell-types for each patient cluster (see Methods). Each patient sample was clustered separately (see patient clustering in Supplementary Fig. 6b) and the clusters are denoted as e.g. "Patient#1-Sample#1-cluster#1" = "Patient 1-S1-C1" (see Supplementary Data 1). **b,** A plot showing the top shared genes between the mouse embryonic RG cluster and the matching patient clusters from the deconvolution analysis in panel (a), the plot shows the genes that are present in the top 100 genes shared between the embryonic RG cluster and at least 15 or more individual patient tumour clusters (see Methods and the full list of shared genes in Supplementary Data 2). The red bars indicate that the gene is in the top 100 genes shared between the patient tumour cluster and the mouse embryonic RG cluster. The numbers on the left indicate the percentage each gene is commonly shared in the total number of patient clusters.
Abbreviations as in Fig. 4.

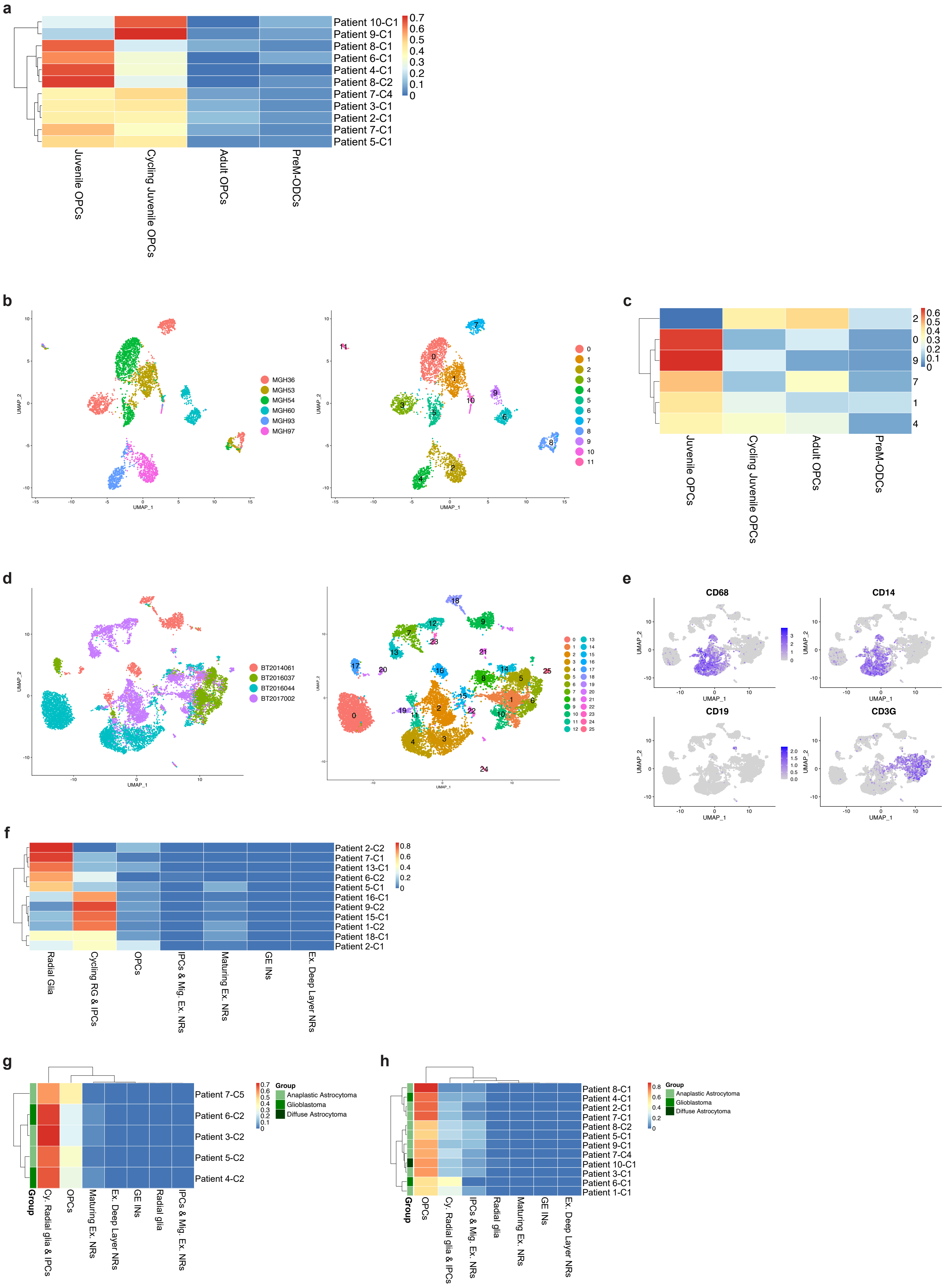

a
Patient 10-C1
Patient 9-C1
Patient 8-C1
Patient 6-C1
Patient 4-C1
Patient 8-C2
Patient 7-C4
Patient 3-C1
Patient 2-C1
Patient 7-C1
Patient 5-C1
Juvenile OPCs
Cycling Juvenile OPCs
Adult OPCs
PreM-ODCs
b
UMAP_1
UMAP_2
MGH36
MGH53
MGH54
MGH60
MGH93
MGH97
c
Juvenile OPCs
Cycling Juvenile OPCs
Adult OPCs
PreM-ODCs
d
BT2014061
BT2016037
BT2016044
BT2017002
e
CD68
CD14
CD19
CD3G
f
Patient 2-C2
Patient 7-C1
Patient 13-C1
Patient 6-C2
Patient 5-C1
Patient 16-C1
Patient 9-C2
Patient 15-C1
Patient 1-C2
Patient 18-C1
Patient 2-C1
Radial Glia
Cycling RG & IPCs
OPCs
IPCs & Mig. Ex. NRs
Maturing Ex. NRs
GE INs
Ex. Deep Layer NRs
g
Patient 7-C5
Patient 6-C2
Patient 3-C2
Patient 5-C2
Patient 4-C2
Group
Anaplastic Astrocytoma
Glioblastoma
Diffuse Astrocytoma
Cy. Radial glia & IPCs
OPCs
Maturing Ex. NRs
Ex. Deep Layer NRs
GE INs
Radial glia
IPCs & Mig. Ex. NRs
h
Patient 8-C1
Patient 4-C1
Patient 2-C1
Patient 7-C1
Patient 8-C2
Patient 5-C1
Patient 9-C1
Patient 7-C4
Patient 10-C1
Patient 3-C1
Patient 6-C1
Patient 1-C1
Group
Anaplastic Astrocytoma
Glioblastoma
Diffuse Astrocytoma
OPCs
Cy. Radial glia & IPCs
IPCs & Mig. Ex. NRs
Radial glia
Maturing Ex. NRs
GE INs
Ex. Deep Layer NRs

Supplementary Fig. 9 | The OPC-matching clusters from IDG and ODG patients map more closely with juvenile rather than adult OPCs. a, Matching of the IDG patient clusters, which showed an OPC-type identity in Fig. 5a, against the cell types identified in the mouse OPC cluster from Supplementary Fig. 5a. **b,** Clustering analysis of scRNA-seq data from 6 ODG patients coloured by the patient identity (left) and distinct clusters (right). This clustering was used in the deconvolution analysis in Fig. 5b. **c,** Matching of the ODG patient clusters, which showed an OPC-type identity in Fig. 5b, against the cell types identified in the mouse OPC cluster from Supplementary Fig. 5a. **d,** Clustering analysis and visualization of scRNA-seq data from 4 supratentorial ependymoma patients. Each dot represents a single cell and the colours correspond to the distinct clusters (left) and the patient identities (right). This clustering was used in the deconvolution analysis in Fig. 5c. **e,** UMAP showing normalized expression levels (colour) of immune cell-specific markers to distinguish the immune clusters/microenvironment from the tumour clusters. **f,** Deconvolution analysis of the IDH-wildtype GBM patient clusters which showed an embryonic RG-type identity in Fig. 4a, against the neuronal/glial cell types identified in the human foetal dataset in Supplementary Fig. 5b. **g,** Matching of the IDG patient clusters, which showed an embryonic RG-type identity in Fig. 5a, against the neuronal/glial cell types identified in the human foetal dataset in Supplementary Fig. 5b. **h,** Matching of the IDG patient clusters, which showed an OPC-type identity in Fig. 5a, against the neuronal/glial cell types identified in the human foetal dataset in Supplementary Fig. 5b.
Abbreviations as in Fig. 4 and Supplementary Fig. 5.

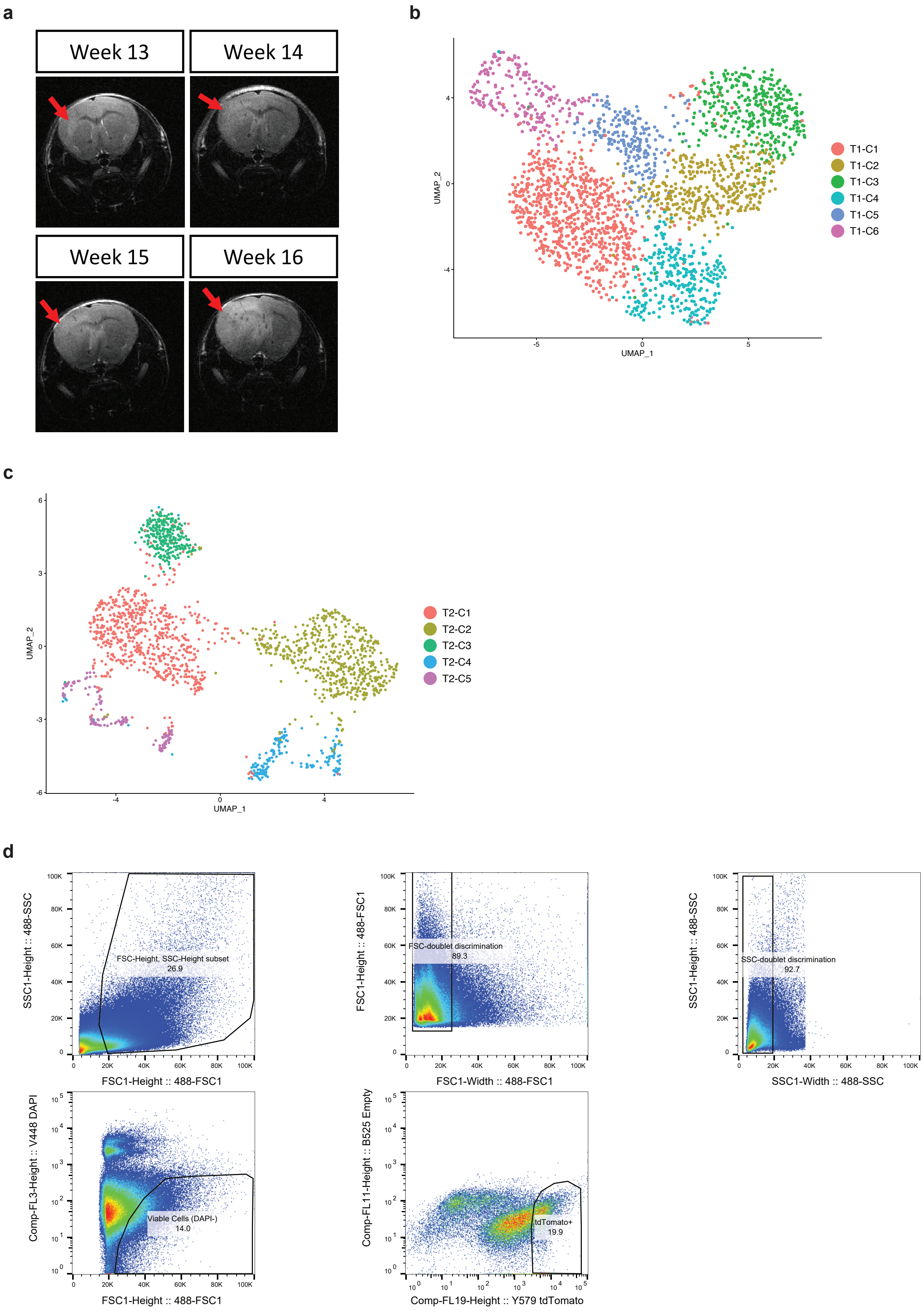
a
Week 13
Week 14
Week 15
Week 16
b
UMAP_2
UMAP_1
T1-C1
T1-C2
T1-C3
T1-C4
T1-C5
T1-C6
c
UMAP_2
UMAP_1
T2-C1
T2-C2
T2-C3
T2-C4
T2-C5
d
SSC1-Height :: 488-SSC
FSC1-Height :: 488-FSC1
FSC-Height, SSC-Height subset
26.9
FSC1-Height :: 488-FSC1
FSC1-Width :: 488-FSC1
FSC-doublet discrimination
89.3
SSC1-Height :: 488-SSC
SSC1-Width :: 488-SSC
SSC-doublet discrimination
92.7
Comp-FL3-Height :: V448 DAPI
FSC1-Height :: 488-FSC1
Viable Cells (DAPI-)
14.0
Comp-FL11-Height :: B525 Empty
Comp-FL19-Height :: Y579 tdTomato
tdTomato+
19.9

Supplementary Fig. 10 | a, MR images of a mutant mouse brain at 13, 14, 15 and 16 weeks of age showing the growth of a tumour encompassing the left hemisphere (red arrows). **b, c,** Clustering analysis of the malignant cells from the mouse tumour replicate 1 (b) and 2 (c), which was used in the deconvolution analysis for Fig. 7. Each dot represents a single cell and colours correspond to the distinct clusters. **d,** FACS plots of the collection strategy of the tdTomato+ cells from the mouse tumour sample in Fig. 7.

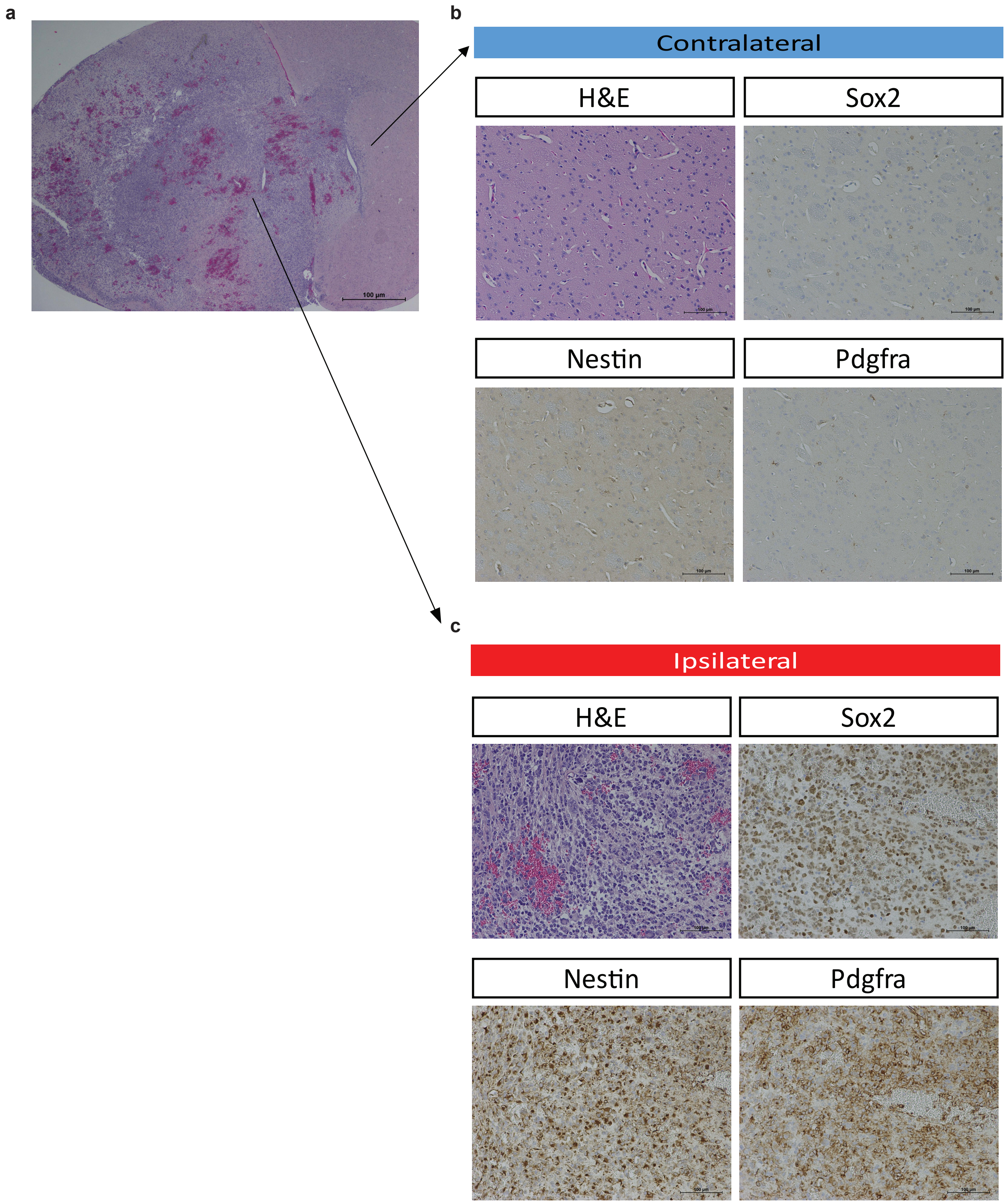

a
b
Contralateral
H&E
Sox2
Nestin
Pdgfra
c
Ipsilateral
H&E
Sox2
Nestin
Pdgfra
100 µm

Supplementary Fig. 11 | a, H&E-stained section of the mouse brain in Fig. 7b. Scale bar, 100μm. **b,** H&E-staining and immunostaining for the three proteins, Sox2, Nestin and Pdgfra, in both the contralateral (top) and tumour/ipsilateral (bottom) hemispheres. Scale bar, 100μm.
