# Supplementary material for: A brain precursor atlas reveals the acquisition of developmental-like states in adult cerebral tumours
Source: Nat Commun. 2022 Jul 19;13:4178. doi: 10.1038/s41467-022-31408-y (PMC9296666; doi:10.1038/s41467-022-31408-y)
Supplement: Supplementary file 2 — Description of Additional Supplementary Files [file 41467_2022_31408_MOESM2_ESM.pdf]

Description of Additional Supplementary Files

File Name: Supplementary Data 1
Description: A summary of the patient IDs related to Figures 4, 5 and Supplementary Fig. 8.

File Name: Supplementary Data 2
Description: Top 100 genes shared between each of the GBM patient tumour clusters and the mouse embryonic RGP cluster related to Supplementary Fig. 8b.
