# Supplementary material for: A brain precursor atlas reveals the acquisition of developmental-like states in adult cerebral tumours
Source: Nat Commun. 2022 Jul 19;13:4178. doi: 10.1038/s41467-022-31408-y (PMC9296666; doi:10.1038/s41467-022-31408-y)
Supplement: Supplementary file 5 — Reporting Summary [file 41467_2022_31408_MOESM5_ESM.pdf]

Corresponding author(s): Lincoln D. Stein, Benjamin D. Simons, Peter B. Dirks

Last updated by author(s): May 20, 2022

Reporting Summary

Nature Research wishes to improve the reproducibility of the work that we publish. This form provides structure for consistency and transparency in reporting. For further information on Nature Research policies, see our Editorial Policies and the Editorial Policy Checklist.

Statistics

For all statistical analyses, confirm that the following items are present in the figure legend, table legend, main text, or Methods section.

n/a	Confirmed	
☐	☒	The exact sample size (*n*) for each experimental group/condition, given as a discrete number and unit of measurement
☐	☒	A statement on whether measurements were taken from distinct samples or whether the same sample was measured repeatedly
☐	☒	The statistical test(s) used AND whether they are one- or two-sided<br>*Only common tests should be described solely by name; describe more complex techniques in the Methods section.*
☐	☒	A description of all covariates tested
☐	☒	A description of any assumptions or corrections, such as tests of normality and adjustment for multiple comparisons
☐	☒	A full description of the statistical parameters including central tendency (e.g. means) or other basic estimates (e.g. regression coefficient) AND variation (e.g. standard deviation) or associated estimates of uncertainty (e.g. confidence intervals)
☐	☒	For null hypothesis testing, the test statistic (e.g. *F*, *t*, *r*) with confidence intervals, effect sizes, degrees of freedom and *P* value noted<br>*Give P values as exact values whenever suitable.*
☒	☐	For Bayesian analysis, information on the choice of priors and Markov chain Monte Carlo settings
☒	☐	For hierarchical and complex designs, identification of the appropriate level for tests and full reporting of outcomes
☒	☐	Estimates of effect sizes (e.g. Cohen's *d*, Pearson's *r*), indicating how they were calculated

Our web collection on statistics for biologists contains articles on many of the points above.

Software and code

Policy information about availability of computer code

Data collection	No software was used
Data analysis	Seurat v3.1.4, biomaRt v2.42.1, pheatmap v1.0.12, ggplot2 v3.3.2, UpsetR v1.4.0, BisqueRNA v1.0.4, CIBERSORT (https://cibersort.stanford.edu), CIBERSORTx (https://cibersortx.stanford.edu), Cell Ranger (v3.1.0, 10x Genomics Inc.), scanpy (v1.5.1), python (v3.6.9), scran (v1.12.1), velocyto (v0.17.17), pySCENIC (v0.10.2), Biorender (https://biorender.com), FlowJo version 10.6.2, MIPAV software (version 8.0.2). The code used for the mouse developmental atlas analysis can be retrieved here: https://github.com/BenSimonsLab/Hamed_Nat-Commun_2022

For manuscripts utilizing custom algorithms or software that are central to the research but not yet described in published literature, software must be made available to editors and reviewers. We strongly encourage code deposition in a community repository (e.g. GitHub). See the Nature Research guidelines for submitting code & software for further information.

Data

Policy information about availability of data

All manuscripts must include a data availability statement. This statement should provide the following information, where applicable:

- Accession codes, unique identifiers, or web links for publicly available datasets
- A list of figures that have associated raw data
- A description of any restrictions on data availability

The raw and processed scRNA-seq data of the mouse developmental atlas and the mouse tumour samples generated in this study have been deposited in the Gene Expression Omnibus (GEO) under accession number (GSE200202). The human IDH-WT GBM1,51, IDH-mutant GBM4, oligodendroglioma3, GSC lines51 and human foetal50 scRNA-seq datasets were obtained from previous publications and are publicly available datasets in GEO, the European Genome-phenome Archive (EGA) and the database of Genotypes and Phenotypes (dbGaP) under the following accession numbers (GSE131928, GSE89567, GSE70630, EGAS00001004656,

phs001836). The human supratentorial ependymoma scRNA-seq data was obtained from the Michael Taylor Lab at SickKids (who deposited the data in EGA under accession number (EGAS00001006237). Source data are provided with this paper. The remaining data are available within the Article, Supplementary Information or Source Data file.

Field-specific reporting

Please select the one below that is the best fit for your research. If you are not sure, read the appropriate sections before making your selection.

- [x] Life sciences
- [ ] Behavioural & social sciences
- [ ] Ecological, evolutionary & environmental sciences

For a reference copy of the document with all sections, see nature.com/documents/nr-reporting-summary-flat.pdf

Life sciences study design

All studies must disclose on these points even when the disclosure is negative.

Sample size	For the mouse developmental single cell dataset: we chose the time-points based on knowledge of the important stages in the process of brain development (embryonic, juvenile and adult) with the goal of collecting 3 time-points or more in each stage. For the mouse tumour samples: we collected the samples at endpoint judged by the magnetic resonance imaging performed weekly. We didn't use any statistical methods to predetermine the sample size.
Data exclusions	No data exclusions
Replication	For the mouse developmental single cell dataset: we collected one replicate per time-point, the early analysis of the data revealed that time-points within the same developmental stage (e.g. juvenile time-points: P0, P2 and P7) show close transcriptional similarities but are different from time-points in another stage, which suggested low batch effects between the time-points. For the mouse tumour samples: we collected two replicates of endpoint tumours.
Randomization	Mice were randomly picked for each experiment.
Blinding	Blinding was not possible in this study.

Reporting for specific materials, systems and methods

We require information from authors about some types of materials, experimental systems and methods used in many studies. Here, indicate whether each material, system or method listed is relevant to your study. If you are not sure if a list item applies to your research, read the appropriate section before selecting a response.

Materials & experimental systems

n/a	Involved in the study	
[ ]	[x]	Antibodies
[x]	[ ]	Eukaryotic cell lines
[x]	[ ]	Palaeontology and archaeology
[ ]	[x]	Animals and other organisms
[ ]	[x]	Human research participants
[x]	[ ]	Clinical data
[x]	[ ]	Dual use research of concern

Methods

n/a	Involved in the study	
[x]	[ ]	ChIP-seq
[ ]	[x]	Flow cytometry
[x]	[ ]	MRI-based neuroimaging

Antibodies

Antibodies used	The following antibodies and dilutions were used: Anti-Sox2 (R&D Cat.# af2018) at 1:150, Anti-Nestin (Novus cat# NB100-1604) at 1:500, Anti-pdgfra (R&D Cat.# af1062) at 1:20, Anti-Goat IgG (H+L), made in horse, biotinylated (Vector labs Cat.# BA-9500) at 1:200 and Anti-chicken IgG (H+L), made in goat, biotinylated (Vector labs cat#BA-9010) at 1:200 dilution.
Validation	All antibodies were validated as shown on the companies websites and have been shown (by the suppliers and in previous publications) to specifically bind to their target proteins.<br>Anti-Sox2 (R&D Cat.# af2018) was used in 138 citations.<br>Anti-Nestin (Novus cat# NB100-1604) was used in 51 citations.<br>Anti-pdgfra (R&D Cat.# af1062) was used in 94 citations.

Animals and other organisms

Policy information about studies involving animals; ARRIVE guidelines recommended for reporting animal research

Laboratory animals	The mouse models used in this study were of mixed strains (C57BL6/129/CD1). We used both males and females in all experiments. The mice were housed in a 12 hour dark/light cycle facility with free access to water and chow. Sox2eGFP mice were provided by Dr. Freda Miller, Toronto, Hospital for Sick Children. Trp53f/f mice were provided by Dr. Chi-chung Hui, Toronto, Hospital for Sick Children. The following transgenic strains were purchased from Jackson Laboratory: NestinCre (JAX# 003771), R26tdTomato (B6.Cg-Gt(ROSA)26Sortm9(CAG-tdTomato)Hze/J) (JAX #007909) and PTENf/f (JAX# 006440). Ages of all the mice used in the various experiments are indicated in the figures.
Wild animals	This study didn't include the use of wild animals
Field-collected samples	This study didn't include field-collected samples
Ethics oversight	All mouse experiments were approved by the Hospital for Sick Children’s Animal Care Committee and following all legal and institutional ethical regulations. AUP #1000044616 approved by the Hospital for Sick Children’s Animal Care Committee.

Note that full information on the approval of the study protocol must also be provided in the manuscript.

Human research participants

Policy information about studies involving human research participants

Population characteristics	Tumour samples were obtained from 4 paediatric patients diagnosed with supratentorial ependymomas. This included 1 male and 3 female patients.
Recruitment	No recruitment strategy as this was based on the availability of samples
Ethics oversight	The human tissue was collected from the Hospital for Sick Children Brain Tumour Tissue Bank after patient consent for banking. The samples were de-identified and the research conducted was performed following informed consent and approval from the Research Ethics Board of the Hospital for Sick Children under REB 1000055059.

Note that full information on the approval of the study protocol must also be provided in the manuscript.

Flow Cytometry

Plots

Confirm that:

- [x] The axis labels state the marker and fluorochrome used (e.g. CD4-FITC).
- [x] The axis scales are clearly visible. Include numbers along axes only for bottom left plot of group (a 'group' is an analysis of identical markers).
- [x] All plots are contour plots with outliers or pseudocolor plots.
- [ ] A numerical value for number of cells or percentage (with statistics) is provided.

Methodology

Sample preparation	Fresh brain tissues from embryo/postnatal Sox2eGFP mice were collected following transcardial perfusion of mice (pregnant females in case of embryo samples) with PBS. The tissue was transferred to a petri dish placed on ice and the cerebrum was isolated under a Leica stereoscope, rinsed in PBS, dissociated into single cells as previously reported4, then passed through a 40-µm mesh cell strainer. Debris was removed using a debris-removal kit® (Miltenyi) and the cells were stained with DAPI followed by sorting the live GFP+ve/-ve cells. For the mouse tumour samples, the mouse harboring the tumour was imaged by MRI 30 minutes before sample collection through transcardial perfusion with PBS and dissection of the hemisphere encompassing the tumour followed by tissue dissociation as described above. Finally, the cells were stained with DAPI before sorting the live tdTomato+ cells.
Instrument	Beckman Coulter MoFlo XDP Cell Sorter: used for sorting all the mouse GFP developmental samples except P13.<br>Sony SH800Z Cell Sorter: used for sorting the GFP P13 developmental time-point.<br>Beckman Coulter MoFlo Astrios Cell Sorter: used for sorting the tdTomato mouse tumour samples.
Software	Data was acquired and cells were sorted on the instruments through the Summit software (version 5.4) and analyzed in FlowJo version 10.6.2
Cell population abundance	Post-sort fractions contained >90% of cells within the gated regions of interest, with some smaller debris in post-sort samples falling outside of the gates on the first plot of scatter characteristics (FSC-height versus SSC-height).
Gating strategy	The gating strategy used in the experiments began by screening based on the scatter properties of the cells on a FSC-height versus SSC-height plot. Subsequently, doublets were screened in the gating strategy by selecting cells with a singular signal

pulse width on a FSC-width versus FSC-height plot. Further doublet discrimination was performed in the gating strategy by selecting cells with a singular signal pulse with on a SSC-width versus SSC-height plot. From single cell gating, a plot of FSC-height versus DAPI (Viability)-log height was used to screen out dead cells by gating on the fraction of DAPI negative, viable cells. Finally, from live single cells, the SOX2-GFP+ cells were selected on a plot of GFP-log height versus log height on an empty channel used to screen out dying cell autofluorescence (B740-log height). Pronounced (over 1 logarithmic decade) differences in GFP fluorescence intensity were used to separate the negatives (<10^3) from the positives (>10^4). Back-gating of the positive and negative fractions was used to ensure the gating hierarchy did not screen out live single cells of interest. Representative plots of the gating strategy are provided in Extended Data Figs. 2b, c.

☒ Tick this box to confirm that a figure exemplifying the gating strategy is provided in the Supplementary Information.
